# Congenital heart disease in England: a national cohort study from fetal diagnosis to end of infancy

**DOI:** 10.1136/heartjnl-2025-326369

**Published:** 2025-10-21

**Authors:** Qi Huang, Rodney C G Franklin, Anna N Seale, Victoria Jowett, Christina Pagel, Sonya Crowe, Katherine L Brown

**Affiliations:** 1 Clinical Operational Research Unit, University College London, London, UK; 2 Paediatric Cardiology, Royal Brompton & Harefield NHS Foundation Trust, London, England, UK; 3 Paediatric Cardiology and Cardiothoracic Surgery, Birmingham Women's and Children's Hospital, Birmingham, England, UK; 4 Institute of Cardiovascular Sciences, University of Birmingham, Birmingham, England, UK; 5 Fetal Cardiology, Great Ormond Street Hospital for Children, London, England, UK; 6 Biomedical Research Centre, Great Ormond Street Hospital for Children, London, England, UK; 7 Institute of Cardiovascular Science, University College London, London, England, UK

**Keywords:** Heart Defects, Congenital, Outcome Assessment, Health Care

## Abstract

**Background:**

Population studies of congenital heart disease (CHD) often include only children receiving cardiac interventions, underestimating the burden of cases without intervention. We evaluated outcomes for all detected structural CHD cases in England from fetal life to the age of 1 year.

**Method:**

We linked the National Congenital Anomaly and Rare Disease Registration Service, the National Congenital Heart Disease Audit, and Office for National Statistics mortality records to construct an incident cohort with estimated delivery/birth dates 2018–2020. Outcomes were: termination of pregnancy, fetal loss (miscarriage/stillbirth), live birth with no cardiac intervention in infancy, and live birth with intervention(s) in infancy. Infant mortality at the age of 1 year was assessed.

**Results:**

Among 11 265 CHD cases, 63.7% were antenatally detected (95% CI 62.8% to 64.6%), rising to 94.2% (92.0% to 96.0%) for hypoplastic left heart syndrome (HLHS). There were 1766 terminations (15.7%, 95% CI 14.7% to 16.7%), 295 fetal losses (2.6%, 95% CI 1.6% to 3.6%), 4538 live births with no infant cardiac intervention (40.3%, 95% CI 39.3% to 41.3%) and 4666 with intervention(s) (41.4%, 95% CI 40.4% to 42.4%). Termination was higher with greater CHD complexity (eg, HLHS 51.1% (95% CI 46.8% to 55.5%) versus isolated ventricular septal defect 6.0% (95% CI 4.3% to 7.7%), p<0.001), non-cardiac comorbidities (23.6% (95% CI 21.9% to 25.4%) vs 11.3% (95% CI 10.1% to 12.6%), p<0.001), and least versus most deprived areas (20.3% (95% CI 17.5% to 23.1%) vs 11.6% (95% CI 9.7% to 13.5%), p<0.001). Infant mortality was 13.3% (602/4538) in the no-intervention group and 5.2% (243/4666) in the intervention group; those deaths without intervention (n=602) were predominantly cases with critical CHD (n=154), preterm birth (n=301) and/or comorbidity (n=362).

**Conclusion:**

This national, linked cohort shows that un-intervened cases account for most infant deaths and that antenatal detection exceeds 90% for the most complex lesions. Registries and quality improvement should include all CHD care pathways to inform counselling and equitable service planning.

**WHAT IS ALREADY KNOWN ON THIS TOPIC**

- Most studies of CHD report only on babies who undergo intervention, which creates bias.

**WHAT THIS STUDY ADDS**

- In England (2018–2020), nearly two-thirds of CHD cases were detected before birth, rising to over 90% for the most complex conditions.
- Outcomes show that 16% of pregnancies ended in termination, 3% in miscarriage or stillbirth, 40% were live births with no intervention in infancy, and 41% were live births that underwent intervention in infancy.
- Around 71% of infant deaths occurred in babies who did not receive intervention.

**HOW THIS STUDY MIGHT AFFECT RESEARCH, PRACTICE OR POLICY**

- Registries and quality improvement efforts should include all CHD cases to provide a complete picture and guide counselling, service planning and policy.

## Introduction

 Many contemporary population or registry-based studies of congenital heart disease (CHD) prevalence^1^ and survival rates^2 3^ are dominated by data sources recording patients who underwent CHD interventions. A small number of studies have tracked the incident cases of structural CHD, based on a combination of those diagnosed in fetal life, a proportion of which end in termination of pregnancy, and CHD cases that were diagnosed postnatally.^4^,^6^ Population-based studies of maternal and perinatal records have reported the prevalence of CHD based on complexity and other important factors.^7^,^9^ However, none of these studies reported a complete picture of CHD cases at the population level, inclusive of (1) All cases diagnosed antenatally alongside their pregnancy outcomes; and (2) All cases with live birth, alongside any occurrence of cardiac intervention(s) and children’s subsequent survival rates to the age of 1 year. This type of cohort analysis is challenging to undertake because it necessitates linkage of complex information from separate sources, compounded by the heterogeneity of CHD, which is described differently across coding schemes and data sources.

Our study was set in the National Health Service (NHS) of England, where healthcare is universal and free of charge at the point of delivery, with fetal anomaly scanning for all pregnancies. Our study questions were:

1. How many and what proportion of structural CHD cases were detected antenatally, by CHD types and by non-cardiac comorbidities?
2. How many and what proportions of incident structural CHD cases had termination of pregnancy, spontaneous fetal loss, live birth followed by no early cardiac intervention and live birth followed by early cardiac intervention?

## Methods

### Study design

We undertook an observational cohort study, based on fetal and procedure-based registries, of structural CHD cases in England. We created a linked data set using the unique patient identifier based on three data sources:

1. National Congenital Anomaly and Rare Disease Registration Service (NCARDRS) (primary data set) consisting of all registered cases of congenital anomalies occurring in babies (including live-born and stillborn), fetal losses and terminations at any gestation in England.^10^ Notification to NCARDRS can occur antenatally, neonatally or from paediatric specialist services.^10^ Anomalies are coded to international standards using the WHO’s International Classification of Diseases 10th Revision (ICD-10).^11^ In this study, we obtained records identified by the European Surveillance of Congenital Anomalies (EUROCAT) as indicative of CHD (any ICD-10 codes ranging from Q20 to Q26^12^) and with an estimated delivery date between 2018 and 2020.
2. National Congenital Heart Disease Audit (NCHDA) (secondary data set) containing all records of cardiac surgical procedures and interventional catheters performed. NCHDA procedure records contain diagnostic and procedure codes from the European Paediatric Cardiac Code (EPCC), derived from the International Paediatric and Congenital Cardiac Code.^13^ We obtained records between September 2017 and March 2022 to determine whether a cardiac intervention was undertaken during infancy and to identify any eligible patients with CHD born during the study period who were not captured in NCARDRS.
3. Office of National Statistics survival status derived from death certifications. This was linked to the NCARDRS extract (in May 2024) and the NCHDA extract (in August 2023).

### Inclusion and exclusion criteria

We included all fetuses and babies with structural CHD in England, with an estimated delivery or birth date between January 2018 and December 2020.

We excluded isolated atrial septal defect and/or patent ductus arteriosus, since these are normal features in utero, and hence cannot in isolation identify structural CHD. We excluded isolated acquired heart disease as beyond the scope of our study.

In NCHDA, we excluded patients with residence outside England or registered as non-NHS because we could not guarantee a complete history.

### Outcome

We ascertained ‘what happened’ to each incident CHD case, categorising these into four distinct ‘healthcare journey types’: (1) Termination of pregnancy, (2) Spontaneous fetal loss (stillbirth or miscarriage), (3) Live birth with no cardiac intervention in infancy (by age 1 year), and (4) Live birth with cardiac intervention(s) in infancy. For live-born babies (3) and (4), we ascertained mortality at the age of 1 year.

### Data management and definitions

**Figure 1:**
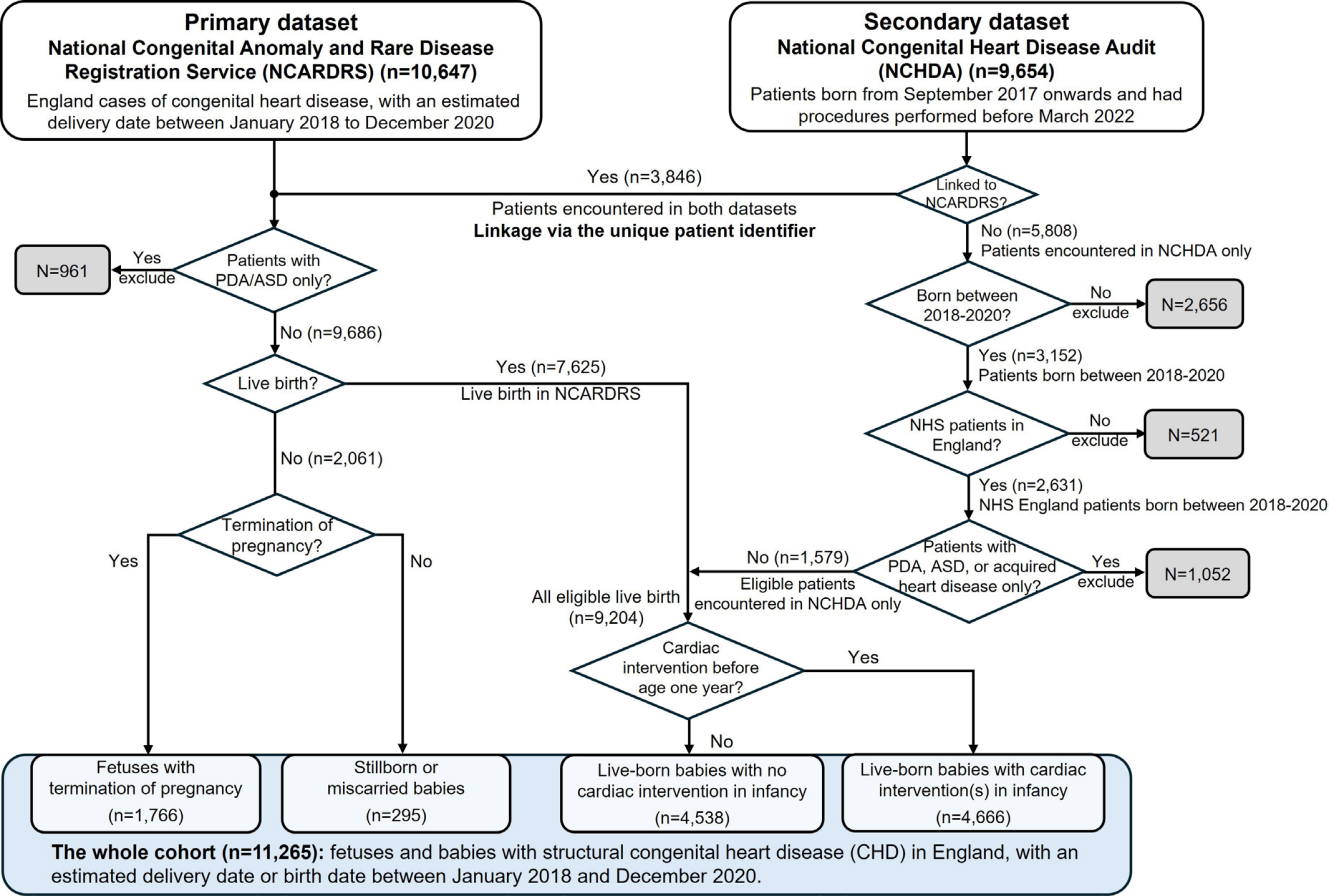
Inclusion and exclusion flow chart and patient journey management. Data linkage was conducted by NHSE (the data controller) using patient identifiers, including NHS number (a unique identifier assigned to everyone at birth in England), last name and postcode. NCARDRS was the primary data set, which was linked to NCHDA to determine whether a cardiac intervention was performed during infancy. We also used NCHDA to identify eligible patients born during the study period who were not included in NCARDRS. ASD, atrial septal defect; NHSE, National Health Service of England; PDA, patent ductus arteriosus.

We created a study cohort of structural CHD cases as depicted in figure 1.

### Antenatal diagnosis

Individual cases were considered to have antenatal detection if the pregnancy was terminated, suspicion or confirmation of anomaly was documented at a gestational age in weeks (this age notation indicates fetal status in NCARDRS), antenatal echocardiography or fetal medicine evaluations were recorded.

### CHD types

We developed a hierarchical allocation algorithm based on either ICD-10 codes (for cases in NCARDRS) or EPCC codes (for cases in NCHDA only) to assign a CHD type (online supplemental tables S1 and S2). We checked the face validity of the allocation algorithm by clinical review of patient histories in the source data, including cross-checks with the assigned NCHDA CHD types,^14^ noting that NCHDA data are validated and benefit from a more detailed coding scheme.

### Demographic and clinical characteristics

We extracted a series of variables to describe patient characteristics: the CHD types, the presence of non-cardiac comorbidities (using EUROCAT subgroups of congenital anomalies),^12^ antenatal detection and prematurity (birth at gestation <37 weeks). We considered patient demographic characteristics of sex, region and socioeconomic status, which was measured using the 2019 Index of Multiple Deprivation (IMD) Scores, divided into five equal quintiles, ranging from 1 (most deprived) to 5 (least deprived).

For patients encountered in both NCARDRS and NCHDA, we prioritised using data from NCARDRS to derive variables, using NCHDA data only when variables were missing in NCARDRS. For patients recorded only in NCHDA, we developed a mapping from EPCC comorbidity codes to EUROCAT subgroups to categorise those with non-cardiac comorbidities (online supplemental table S3).

Region was not available in our NCHDA extract and hence we assigned region based on the treating centre for cases that only appeared in NCHDA, meaning that we had to merge certain regions that could not be identified reliably (online supplemental table S4).

### Descriptive analysis

We calculated the number (%) of incident structural CHD cases with antenatal detection and the number (%) of incident structural CHD cases that followed each distinct healthcare journey, with 95% CIs calculated using binomial exact method and multinomial Sison and Glaz method, respectively. Comparisons between levels of demographic and clinical characteristics were tested using χ^2^ tests.

For live-born babies, we calculated the number (%) infant mortality rate.

We applied data disclosure control rules that suppress all counts from 1 to 5, allowing 0 count to be shown.

### Missing data

There were missing data for sex (590 (5.2%)), mainly in fetuses with termination of pregnancy (557), deprivation (51 (0.5%)) and survival status at 1 year (65 (0.7%) of 9204 live births). We conducted complete data analysis.

### Patient and public involvement

At the request of patient and public advisors, and in collaboration with the charity *Little Hearts Matter*,^15^ we developed diagrams illustrating patient journeys and outcomes from fetal life to 1 year of age to facilitate communication and antenatal counselling for families. These were created for the CHDs: hypoplastic left heart syndrome (HLHS), non-HLHS functionally univentricular heart (FUH), transposition of the great arteries (TGA), aortic stenosis and pulmonary atresia based on their good match between ICD-10 and EPCC coding schemes, and family viewpoints as to their importance in a prior study.^2^

Data management and analyses were performed with Stata (V.15) and R (V.4.4.0).

## Results

### Cohort population

We depict the linkages, inclusions and exclusions in the flow chart (figure 1): there were 9686 cases who met inclusion criteria from NCARDRS, and a further 1579 patients who met inclusion criteria recorded only in NCHDA, creating a cohort of 11 265 structural CHD cases. Of all 9204 eligible live births with CHD, 3716 appeared in both data sets.

We show the number of cases for each CHD type in online supplemental table S5. There were 5749 (51.0%) boys and 3983 (35.4%) that had a non-cardiac comorbidity. Residence in a deprived neighbourhood was most common (quintiles 1 (most deprived) to 5 (least deprived): 3185 (28.3%), 2618 (23.2%), 2135 (19.0%), 1771 (15.7%), 1505 (13.4%), 51 (0.5%) missing). Among 9204 live-born babies, 2339 (25.4%) were preterm.

### Antenatal detection

Of 11 265 cases, 7173 (63.7% (95% CI 62.8% to 64.6%)) had antenatal detection, with significant variation by CHD type (table 1). The highest rates (>90%) were in HLHS: 94.2% (95% CI 92.0% to 96.0%); non-HLHS FUH: 95.4% (95% CI 92.4% to 97.5%); congenitally corrected transposition: 97.2% (95% CI 90.2% to 99.7%); atrial isomerism: 94.7% (95% CI 90.2% to 97.6%); common arterial trunk: 91.5% (95% CI 85.7% to 95.6%); and double outlet ventricle: 93.9% (95% CI 90.4% to 96.4%). The lowest rates (<50%) were in aortic stenosis: 39.9% (95% CI 32.4% to 46.0%); totally anomalous pulmonary venous connection (TAPVC): 18.9% (95% CI 13.0% to 26.2%); pulmonary stenosis: 26.8% (95% CI 23.0% to 30.9%); non-obstructive mitral-aortic valve diseases: 30.0% (95% CI 24.5% to 36.0%); other pulmonary anomalies: 39.0% (95% CI 32.4% to 46.0%); and isolated ventricular septal defect (VSD): 44.6% (95% CI 42.8% to 46.4%). Antenatal diagnosis was more likely with non-cardiac comorbidities than without: 75.8% (95% CI 74.5% to 77.2%) vs 57.0% (95% CI 55.9% to 58.2%), p<0.001; and with full-term birth than preterm birth 57.8% (95% CI 56.6% to 59.0%) vs 50.0% (95% CI 48.0% to 52.1%), p<0.001 (online supplemental table S6). The highest antenatal diagnosis rate was in the south-east of England 69.0% (95% CI 67.6% to 70.3%) and the lowest rate in the Northern region 53.8% (95% CI 49.7% to 58.0%), p<0.001; and there were slightly lower rates with residence in the most deprived quintile 62.1% (95% CI 60.4% to 63.8%) versus least deprived quintile 66.1% (95% CI 63.7% to 68.5%), p=0.04.

**Table 1:** The numbers and proportions of antenatal detection by diagnosis types

	Number of cases	Number of cases with antenatal detection	Antenatal detection rate with 95% CI
**Whole cohort**	11 265	7173	63.7% (62.8% to 64.6%)
**By congenital heart disease (CHD) diagnosis types (in order of decreasing complexity**)			
Hypoplastic left heart syndrome (HLHS)	556	524	94.2% (92.0% to 96.0%)
Functionally univentricular heart (FUH) non-HLHS	303	289	95.4% (92.4% to 97.5%)
Congenitally corrected transposition	71	69	97.2% (90.2% to 99.7%)
Atrial isomerism with complex CHD	171	162	94.7% (90.2% to 97.6%)
Common arterial trunk	142	130	91.5% (85.7% to 95.6%)
Transposition of the great arteries (TGA, all types)	533	459	86.1% (82.9% to 88.9%)
Aortic stenosis (valvar, subvalvar)	326	130	39.9% (34.5% to 45.4%)
Pulmonary atresia (intact septum or with ventricular septal defect (VSD))	273	240	87.9% (83.4% to 91.5%)
Tetralogy of Fallot (all types)	794	620	78.1% (75.0% to 80.9%)
Double outlet ventricle (miscellaneous types)	278	261	93.9% (90.4% to 96.4%)
Atrioventricular septal defect (AVSD, all types)	907	738	81.4% (78.7% to 83.9%)
Totally anomalous pulmonary venous connection (TAPVC)	148	28	18.9% (13.0% to 26.2%)
Aortic obstruction	848	483	57.0% (53.5% to 60.3%)
Aortic arch malformations + vascular ring	842	757	89.9% (87.7% to 91.9%)
Tricuspid valve abnormality (including Ebstein’s)	315	177	56.2% (50.5% to 61.7%)
Pulmonary stenosis	507	136	26.8% (23.0% to 30.9%)
Non-obstructive mitral-aortic valve diseases	260	78	30.0% (24.5% to 36.0%)
Pulmonary other group: valve non-obstructive or pulmonary artery diseases	210	82	39.0% (32.4% to 46.0%)
Isolated VSD	2981	1330	44.6% (42.8% to 46.4%)
Other ungrouped CHDs	800	480	60.0% (56.5% to 63.4%)

### Healthcare journeys

**Figure 2:**
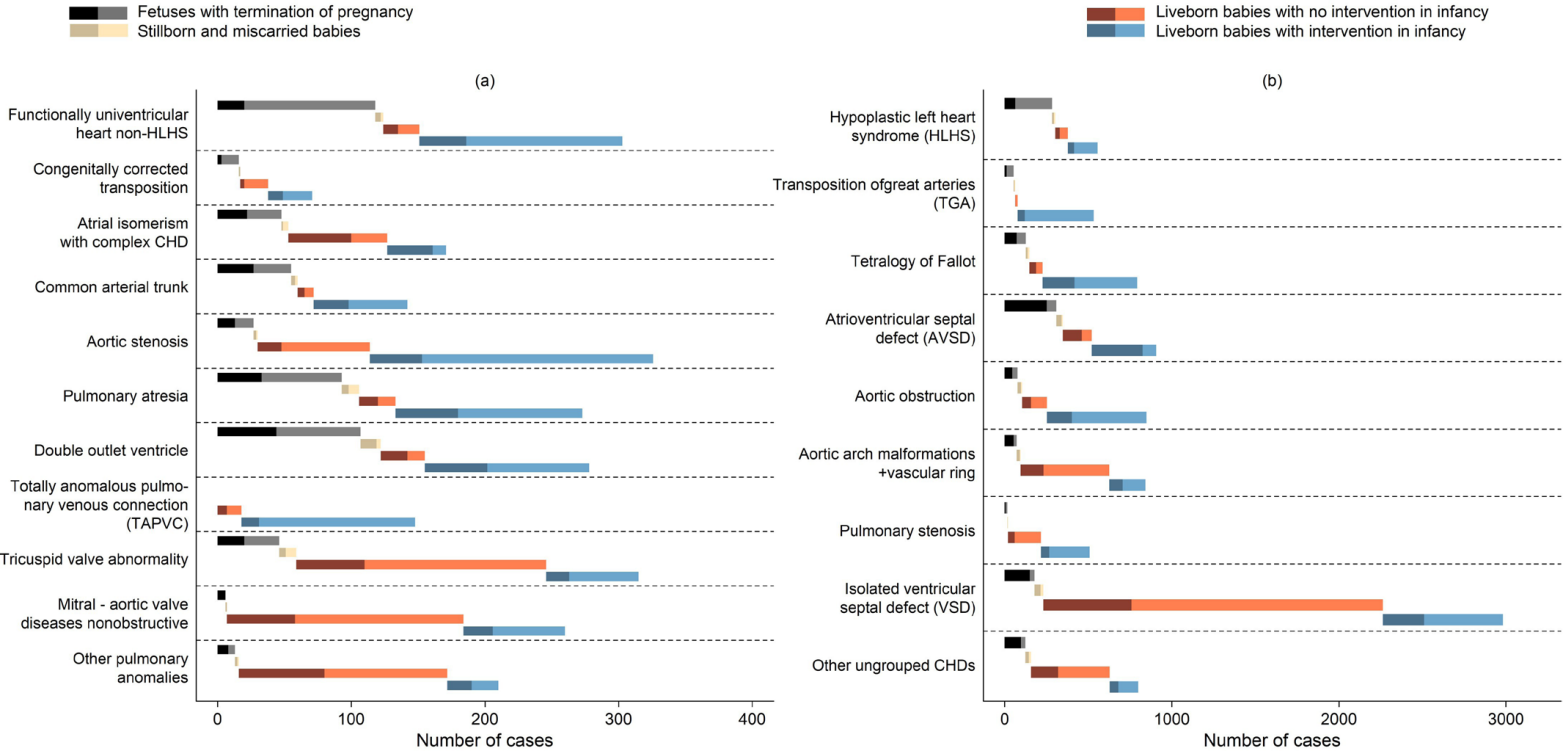
Number and proportion of structural CHD cases in different healthcare journeys by diagnosis types, with cases with comorbidities shown in dark colours. Panel (**a**) Diagnoses with number of cases <400. (**b**) Diagnoses with number of cases >400.

Of the whole cohort, there were 1766 (15.7% (95% CI 14.7% to 16.7%)) fetuses with termination of pregnancy, 295 (2.6% (95% CI 1.6% to 3.6%)) stillborn or miscarried babies, 4538 (40.3% (95% CI 39.3% to 41.3%)) live-born babies with no cardiac intervention in infancy and 4666 (41.4% (95% CI 40.4% to 42.4%)) live-born babies who underwent at least one cardiac intervention during infancy. Table 2 shows the distribution of healthcare journeys by diagnosis type and figure 2 illustrates the breakdown by comorbidity (data in online supplemental table S7). Variations by level of deprivation, region, CHD type and presence of comorbidities are reported in online supplemental table S8.

**Table 2:** The distribution of patient healthcare journeys by diagnosis types

	Number of cases	Number of cases that followed each distinct healthcare journey, along with the corresponding rate and 95% CI			
		Fetuses with termination of pregnancy	Stillborn or miscarried babies	Live-born babies who had no cardiac intervention in infancy	Live-born babies who underwent cardiac intervention(s) in infancy
**Whole cohort**	11 265	1766; 15.7% (14.7% to 16.7%)	295; 2.6% (1.6% to 3.6%)	4538; 40.3% (39.3% to 41.3%)	4666; 41.4% (40.4% to 42.4%)
**By congenital heart disease (CHD) diagnosis types (in order of decreasing complexity**)					
Hypoplastic left heart syndrome (HLHS)	556	284; 51.1% (46.8% to 55.5%)	18; 3.2% (0% to 7.7%)	76; 13.7% (9.4% to 18.1%)	178; 32.0% (27.7% to 36.4%)
Functionally univentricular heart (FUH) non-HLHS	303	118; 38.9% (33.3% to 45.1%)	6; 2.0% (0% to 8.2%)	27; 8.9% (3.3% to 15.1%)	152; 50.2% (44.6% to 56.3%)
Congenitally corrected transposition	71	16; 22.5% (11.3% to 35.5%)	*	21; 29.6% (18.3% to 42.5%)	33; 46.5% (35.2% to 59.4%)
Atrial isomerism with complex CHD	171	48; 28.1% (20.5% to 36.4%)	5; 2.9% (0% to 11.3%)	74; 43.3% (35.7% to 51.6%)	44; 25.7% (18.1% to 34.1%)
Common arterial trunk	142	55; 38.7% (30.3% to 47.3%)	5; 3.5% (0% to 12.1%)	12; 8.5% (0.0% to 17.0%)	70; 49.3% (40.8% to 57.9%)
Transposition of the great arteries (TGA, all types)	533	53; 9.9% (7.3% to 12.9%)	9; 1.7% (0% to 4.7%)	15; 2.8% (0.2% to 5.8%)	456; 85.6% (82.9% to 88.5%)
Aortic stenosis (valvar, subvalvar)	326	27; 8.3% (3.4% to 13.8%)	*	84; 25.8% (20.9% to 31.2%)	212; 65.0% (60.1% to 70.5%)
Pulmonary atresia (intact septum or with VSD)	273	93; 34.1% (28.2% to 40.5%)	13; 4.8% (0% to 11.2%)	27; 9.9% (4.0% to 16.4%)	140; 51.3% (45.4% to 57.7%)
Tetralogy of Fallot (all types)	794	126; 15.9% (12.8% to 19.0%)	21; 2.6% (0% to 5.8%)	79; 9.9% (6.9% to 13.1%)	568; 71.5% (68.5% to 74.7%)
Double outlet ventricle (miscellaneous types)	278	107; 38.5% (32.4% to 44.8%)	15; 5.4% (0% to 11.7%)	33; 11.9% (5.8% to 18.2%)	123; 44.2% (38.1% to 50.6%)
Atrioventricular septal defect (AVSD, all types)	907	308; 34.0% (30.5% to 37.6%)	41; 4.5% (1.1% to 8.1%)	173; 19.1% (15.7% to 22.7%)	385; 42.4% (39.0% to 46.1%)
Totally anomalous pulmonary venous connection (TAPVC)	148	0; 0%	0; 0%	18; 12.2% (7.4% to 17.2%)	130; 87.8% (83.1% to 92.9%)
Aortic obstruction	848	76; 9.0% (6.0% to 12.1%)	29; 3.4% (0.5% to 6.5%)	148; 17.5% (14.5% to 20.6%)	595; 70.2% (67.2% to 73.3%)
Aortic arch malformations + vascular ring	842	71; 8.4% (5.2% to 11.9%)	24; 2.9% (0% to 6.3%)	531; 63.1% (59.9% to 66.5%)	216; 25.7% (22.4% to 29.1%)
Tricuspid valve abnormality (including Ebstein’s)	315	46; 14.6% (9.2% to 20.1%)	13; 4.1% (0% to 9.7%)	187; 59.4% (54.0% to 64.9%)	69; 21.9% (16.5% to 27.4%)
Pulmonary stenosis	507	16; 3.2% (0.0% to 7.7%)	*	196; 38.7% (34.3% to 43.2%)	291; 57.4% (53.1% to 62.0%)
Non-obstructive mitral-aortic valve diseases	260	6; 2.3% (0.0% to 8.3%)	*	177; 68.1% (62.7% to 74.1%)	76; 29.2% (23.8% to 35.2%)
Pulmonary other group—valve non-obstructive or pulmonary artery diseases	210	13; 6.2% (1.0% to 12.3%)	*	156; 74.3% (69.0% to 80.4%)	38; 18.1% (12.9% to 24.2%)
Isolated ventricular septal defect (VSD)	2981	179; 6.0% (4.3% to 7.7%)	52; 1.7% (0.1% to 3.5%)	2,031; 68.1% (66.5% to 69.9%)	719; 24.1% (22.4% to 25.8%)
Other ungrouped CHDs	800	124; 15.5% (12.1% to 19.1%)	32; 4.0% (0.6% to 7.6%)	473; 59.1% (55.8% to 62.7%)	171; 21.4% (18.0% to 24.9%)

Chi-square test for comparing pathway journeys among different CHD diagnosis types, p<0.001.

* Sample size 1–5 (number suppressed).

### Termination of pregnancy

Termination rates varied widely by CHD type, with the highest proportion of 51.1% (95% CI 46.8% to 55.5%) for HLHS and 38.9% (95% CI 33.3% to 45.1%) for non-HLHS FUH (table 2 and figure 2). Termination was more common in cases with non-cardiac comorbidity than without, at 23.6% (95% CI 21.9% to 25.4%) vs 11.3% (95% CI 10.1% to 12.6%), p<0.001. Highest rates of non-cardiac comorbidities were with terminations for atrioventricular septal defect (AVSD) 81.5% (251/308) and isolated VSD 83.2% (149/179). The termination rate varied by region, being lowest: 12.5% (95% CI 9.5% to 15.5%) in the north-west and highest: 18.7% (95% CI 14.5% to 23.1%) in Wessex, p<0.001, and was nearly twice as high with maternal residence in the least deprived compared with the most deprived areas: 20.3% (95% CI 17.5% to 23.1%) vs 11.6% (95% CI 9.7% to 13.5%), p<0.001.

### Fetal loss

The number of pregnancies affected by miscarriage and stillbirth was low, with small numbers for individual CHD types and regions. The rate of fetal loss was higher with non-cardiac comorbidity: 4.8% (95% CI 3.1% to 6.6%) than without: 1.4% (95% CI 0.2% to 2.7%), p<0.001; and with residence in the most deprived areas: 3.6% (95% CI 1.8% to 5.5%) compared with least deprived areas: 1.7% (95% CI 0% to 4.5%), p<0.001.

### Live births

**Figure 3:**
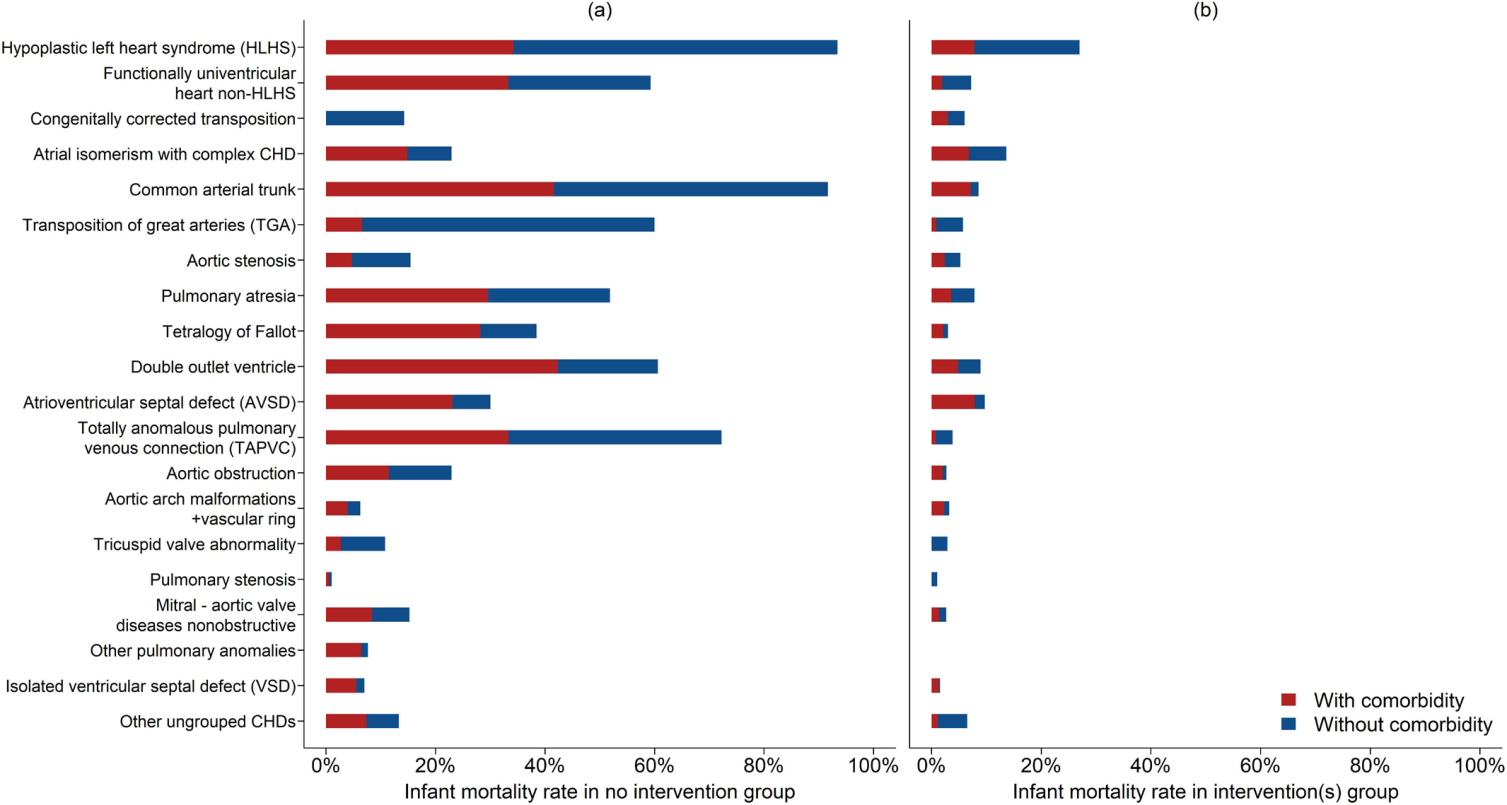
Infant mortality rates (death before the age of 1 year) for live-born babies with CHD by diagnosis types, with a breakdown by the presence of non-cardiac comorbidities. Infant mortality rate in panel (**a**): patients with no intervention in infancy, and (**b**) patients with intervention(s) in infancy. CHD, congenital heart disease.

Of 9204 live births, 4538 (49.3% (95% CI 48.3% to 50.3%)) had no cardiac intervention, among whom 13.3% (602/4538) died in infancy; and 4666 (50.7% (95% CI 49.7% to 51.7%)) had cardiac intervention(s), among whom 5.2% (243/4666) died in infancy. In figure 3 (data in online supplemental table S9) we display infant mortality rates for live births by CHD type (affected by non-cardiac comorbidities in red and isolated CHD in blue); with panel (a) depicting the no-intervention group and panel (b) the intervention(s) group. Among the no-intervention group, the majority of those with HLHS, non-HLHS FUH, common arterial trunk, TGA, pulmonary atresia, double outlet ventricle and TAPVC died in infancy, accounting for 154/602 (25.6%) deaths.

We show the 9204 live births divided into those who had no cardiac intervention and those who had cardiac intervention(s) by demographic and clinical factors in online supplemental table S10, and we show infant mortality rates for the two groups by each demographic and clinical factor in online supplemental table S11. Those with preterm live birth were more likely to have no intervention: 62.2% (95% CI 60.2% to 64.1%) vs 44.9% (95% CI 43.7% to 46.1%) of those with full term birth, p<0.001. Preterm babies accounted for 50% (301/602) deaths in the ‘no intervention’ group. The proportions of live births with non-cardiac comorbidities were similar between the no intervention/intervention(s) groups, 48.7% (95% CI 46.9% to 50.6%) vs 49.6% (95% CI 48.3% to 50.8%) although specific individual comorbidity rates varied. Babies with comorbidities accounted for 362/602 (60.1%) deaths in the ‘no intervention’ group. Among live births, boys were less likely to be in the ‘no intervention’ group than girls: 45.9% (95% CI 44.5% to 47.3%) vs 53.3% (95% CI 51.8% to 54.8%), p<0.001. There were no clear differences in the proportions of who did not have/did have intervention(s) based on neighbourhood deprivation.

### Patient journey and outcome diagrams

**Figure 4:**
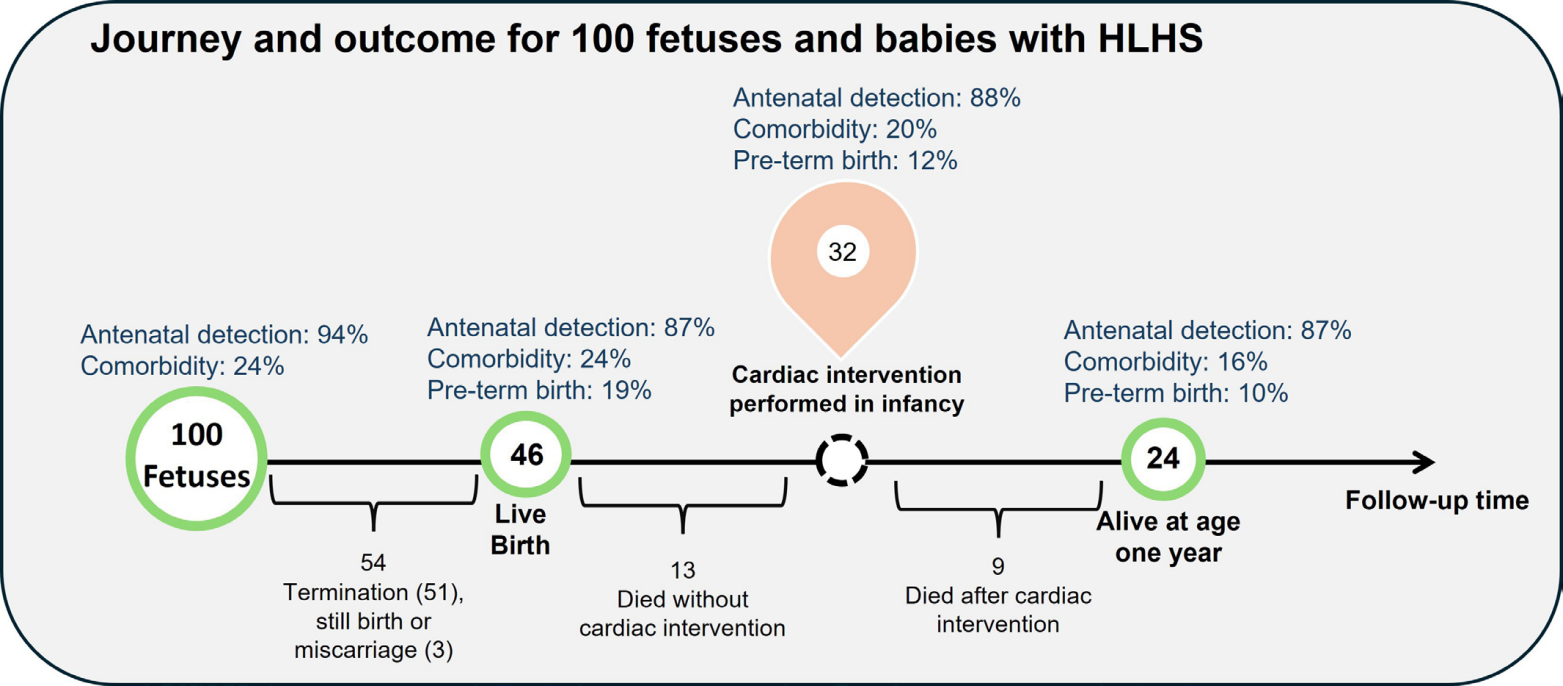
Journey diagram for fetuses and babies with hypoplastic left heart syndrome (HLHS). In our data set, 1 in 100 HLHS cases survived to age 1 year without intervention.

As shown in figure 4, for every 100 fetuses and babies with HLHS, after termination of pregnancy and fetal losses, 46 were live-born and 24 survived to the age of 1 year. Of 46 live-born babies, 13 died without intervention and 32 underwent a cardiac intervention. Patients born preterm or with comorbidities were less likely to survive beyond their first year, that is, the rate of preterm birth and comorbidities decreased from 19% and 24% among all live births to 10% and 16%, respectively, among survivors at 1 year. We present journey and outcome diagrams for non-HLHS FUH, TGA, aortic stenosis and pulmonary atresia in online supplemental figure S1.

## Discussion

### Summary and implications of findings

We present a novel population-based cohort study of incident cases of structural CHD in England, using linked routinely collected data sets. We found that antenatal detection occurred for 6 of 10 cases, rising to 9 of 10 cases of very complex CHD. The rate of pregnancy termination was only 15.7%; with the highest termination rate for HLHS, followed by non-HLHS FUH. Our study is one of the first to follow all detected cases during infancy, irrespective of whether a cardiac intervention was undertaken, finding that 7 out of 10 deaths in infancy occurred in the ‘no-intervention group’. For critical CHDs, early intervention is necessary to maintain life. Hence, the babies with critical CHDs (eg, HLHS) who had no intervention are likely to have received comfort care based on parental choice, or because they were too unwell, or too complex for intervention(s) to be undertaken. For CHDs of lesser severity (eg, VSD) and no other complicating factors, the reason for no intervention most likely reflects clinical judgement that intervention was unnecessary during infancy. Alternatively, a no-intervention pathway may have been determined by a child’s complexity from comorbidities and/or preterm birth. The relationship between social characteristics and the management pathway of no-intervention in CHD is poorly understood and requires further research. We have presented graphical displays of our findings for key CHD types, in a format designed for families, receiving positive feedback from the charity *Little Hearts Matter*.

### Context

Between 2018 and 2020, there were 1822 053 live births in England.^16^ Of 11 265 structural CHD cases in our study, 8 out of 10 were live-born, giving a detected incidence of 5.1 cases per 1000 live births, and 2.3 per 1000 live births for major CHD per EUROCAT^17^ (4114/9204 (44.7%)), which is in line with other sources.^4 6 18^ A report from regional birth defect surveillance programmes from 12 countries in Europe, America and Asia reported variation in antenatal detection by participating region.^19^ Antenatal detection rates have improved over time in the UK,^20^ and our data reflect a recent period. The ‘12 countries study’ found high antenatal detection rates for HLHS (64% compared with 94.2% in England) and lowest antenatal rates for TAPVC (28% compared with 18.9% in England), and for the CHDs that are not as easily seen on a four-chamber fetal ultrasound scan (eg, coarctation) or may evolve over time (eg, aortic or pulmonary stenosis).^19^

Analysis of 3.3 million live births between 1991 and 2021 in the Czech Republic found higher pregnancy termination rates, for example, HLHS 86% and AVSD 68% in the Czech Republic^4^ versus 51.1% and 34.5% in England. A Danish population-based study found increasing termination rates over time, with a rate of 39.1% in 2013.^18^ Conversely, a recent study from Utah, USA, reported a lower termination rate of 4.0% (131/3251) and 5.0% (164/3251) fetal loss.^21^ The explanation of differences in pregnancy termination or abstention from CHD interventions by region is complex but involves both cultural factors and the conduct of healthcare systems. We note that children in general in England are more likely to live in deprived areas: in our study period, the residences of families with any live birth by IMD quintiles were 25.8% (most deprived), 22.4%, 19.5%, 17.2% and 15.2% (least deprived).^16^ Families with a baby affected by CHD were even more likely to reside in deprived neighbourhoods. Selected international studies also found a higher prevalence of CHD with socioeconomic deprivation^522^,^24^ and the drivers of this require further research. Moreover, English adults of minoritised ethnic background have poorer socioeconomic circumstances than adults with white ethnic backgrounds,^25^ and families with Asian and black backgrounds are more likely than white families to have a child affected by CHD.^26 27^ We were unable to include ethnicity data in our study, and hence were unable to explore this possible contributor to the findings. Although termination of pregnancy was the least likely for families residing in the most deprived areas, we were not able to determine the extent to which this explains our findings, thus motivating further research. We note that stillbirths are more common in areas of high deprivation compared with low deprivation in England,^28^ which is in line with our findings. The observed link between female sex and a greater likelihood of no intervention with CHD requires further research. We note similar poorer outcomes among the intervened population,^29 30^ which might potentially relate to smaller size or greater case complexity in girls.

### Limitations

Our study is limited by the data quality in NCARDRS, which relies on those with a range of training backgrounds and prenatal images, potentially undermining the accuracy of assigned CHD type. We note that 1579 cases, 80.3% with postnatal diagnosis, were ascertained from NCHDA and not NCARDRs, meaning these were missed by NCARDRS, and one explanation is later detection. Cases diagnosed only after death, that is, CHD cases only represented on death certificates, were not captured by our data sources.

We used the ICD-10 coding scheme to assign a CHD type based on a hierarchy (ie, in order of clinical severity) using a newly developed algorithm. We chose to include all CHD types for which cardiac surgery is undertaken in infancy, given the clinical importance of this intervention. We excluded atrial septal defect and isolated patent ductus arteriosus since these cannot be interpreted as CHD in fetal life, and the postnatally diagnosed cases in our study were derived from the interventional registry: both methods will have reduced the number of cases. We reported our derived CHD incidence using EUROCAT major CHD criteria to aid interpretation of our findings.

## Conclusions

Our contemporary population-based analysis of linked health records enabled the ascertainment and evaluation of all newly detected structural CHD cases, antenatally to the age of 1 year, and emphasises the importance of considering cases with no intervention, among whom most deaths occurred. Our data may be used to inform families and should motivate further research into care pathways for fetuses and babies affected by CHD, especially un-intervened children.

## Supplementary material

10.1136/heartjnl-2025-326369 online supplemental file 1

## Data Availability

No data are available.

## References

[1] Gilboa SM, Devine OJ, Kucik JE (2016). Congenital heart defects in the United States: estimating the magnitude of the affected population in 2010. Circulation.

[2] Brown KL, Huang Q, Espuny-Pujol F (2024). Evaluating Long-Term Outcomes of Children Undergoing Surgical Treatment for Congenital Heart Disease for National Audit in England and Wales. J Am Heart Assoc.

[3] Öztürk A-G, Dellborg M, Damlin A (2024). Long-term survival in patients with univentricular heart: A nationwide, register-based cohort study. *Int J Cardiol Congenit Heart Dis*.

[4] Tomek V, Jicínská H, Pavlícek J (2023). Pregnancy Termination and Postnatal Major Congenital Heart Defect Prevalence After Introduction of Prenatal Cardiac Screening. *JAMA Netw Open*.

[5] Miao Q, Dunn S, Wen SW (2023). Association between maternal marginalization and infants born with congenital heart disease in Ontario Canada. BMC Public Health.

[6] Lytzen R, Vejlstrup N, Bjerre J (2018). Live-Born Major Congenital Heart Disease in Denmark: Incidence, Detection Rate, and Termination of Pregnancy Rate From 1996 to 2013. JAMA Cardiol.

[7] Dolk H, Loane M, Garne E (2011). European Surveillance of Congenital Anomalies Working G. Congenital heart defects in Europe: prevalence and perinatal mortality, 2000 to 2005. Circulation.

[8] Mamasoula C, Addor M-C, Carbonell CC (2022). Prevalence of congenital heart defects in Europe, 2008-2015: A registry-based study. Birth Defects Res.

[9] Khoshnood B, Lelong N, Houyel L (2012). Prevalence, timing of diagnosis and mortality of newborns with congenital heart defects: a population-based study. Heart.

[10] Broughan JM, Wreyford B, Martin D (2024). Cohort profile: the National Congenital Anomaly Registration Dataset in England. BMJ Open.

[11] World Health Organization International statistical classification of diseases and related health problems10th revision. https://icd.who.int/browse10/2019/en.

[12] European surveillance of congenital anomalies (eurocat) guide 1.4: instruction for the registration of congenital anomalies. https://eu-rd-platform.jrc.ec.europa.eu/system/files/public/JRC-EUROCAT-Full%20Guide%201%204%20version%2022-Nov-2021.pdf.

[13] International paediatric and congenital cardiac code. https://ipccc.net/download-the-ipccc.

[14] Brown KL, Rogers L, Barron DJ (2017). Incorporating Comorbidity Within Risk Adjustment for UK Pediatric Cardiac Surgery. Ann Thorac Surg.

[15] Little hearts matter. https://www.lhm.org.uk.

[16] (2015). Live births by month of occurrence and imd decile. https://www.ons.gov.uk/peoplepopulationandcommunity/birthsdeathsandmarriages/livebirths/adhocs/1703livebirthsbymonthofoccurrenceandimddecileenglandandwales2015to2022.

[17] Bergman JEH, Perraud A, Barišić I (2024). Updated EUROCAT guidelines for classification of cases with congenital anomalies. Birth Defects Res.

[18] Leirgul E, Fomina T, Brodwall K (2014). Birth prevalence of congenital heart defects in Norway 1994-2009--a nationwide study. Am Heart J.

[19] Bakker MK, Bergman JEH, Krikov S (2019). Prenatal diagnosis and prevalence of critical congenital heart defects: an international retrospective cohort study. BMJ Open.

[20] (2020). National congenital heart disease audit (NCHDA) 2020 summary report (2018/19 data). https://www.nicor.org.uk/national-cardiac-audit-programme/previous-reports/congenital-heart-disease-2/2020-1/national-congenital-heart-disease-audit-nchda-final?layout=file.

[21] Jepson BM, Metz TD, Miller TA (2023). Pregnancy loss in major fetal congenital heart disease: incidence, risk factors and timing. Ultrasound Obstet Gynecol.

[22] Miao Q, Dunn S, Wen SW (2021). Neighbourhood maternal socioeconomic status indicators and risk of congenital heart disease. BMC Pregnancy Childbirth.

[23] Peyvandi S, Baer RJ, Chambers CD (2020). Environmental and Socioeconomic Factors Influence the Live-Born Incidence of Congenital Heart Disease: A Population-Based Study in California. J Am Heart Assoc.

[24] Li X, Sundquist J, Hamano T (2016). Neighbourhood Deprivation, Individual-Level and Familial-Level Socio-demographic Factors and Risk of Congenital Heart Disease: A Nationwide Study from Sweden. Int J Behav Med.

[25] UK population by ethnicity and socioeconomic status. https://www.ethnicity-facts-figures.service.gov.uk/uk-population-by-ethnicity/demographics/socioeconomic-status/latest/#:~:text=Summary%20of%20Socioeconomic%20status%20Socio,other’%20ethnic%20group%20(1.0%25).

[26] Knowles RL, Ridout D, Crowe S (2017). Ethnic and socioeconomic variation in incidence of congenital heart defects. Arch Dis Child.

[27] Hadjicosta E, Franklin R, Seale A (2022). Cohort study of intervened functionally univentricular heart in England and Wales (2000-2018). Heart.

[28] Birth characteristics in england and wales: 2022. https://www.ons.gov.uk/peoplepopulationandcommunity/birthsdeathsandmarriages/livebirths/bulletins/birthcharacteristicsinenglandandwales/2022.

[29] Knowles RL, Ridout D, Huang Q (2024). Influence of Sex, Race and Ethnicity, and Deprivation on Survival and Completion of the Fontan Pathway for Children With Functionally Single Ventricle Heart Disease. Circulation.

[30] Seifert HA, Howard DL, Silber JH (2007). Female gender increases the risk of death during hospitalization for pediatric cardiac surgery. J Thorac Cardiovasc Surg.

