# Supplementary material for: Congenital heart disease in England: a national cohort study from fetal diagnosis to end of infancy
Source: Heart. 2025 Oct 21;112(9):e326369. doi: 10.1136/heartjnl-2025-326369 (PMC13151507; doi:10.1136/heartjnl-2025-326369)

# Supplemental materials for the manuscript: Population-based analysis of journeys taken by incident cases of structural congenital heart diseases from fetal life through infancy in England.

## Supplementary Table S1: Hierarchical mapping from International Classification of Diseases 10th Revision (ICD-10) codes to CHD diagnosis types.

| **ICD-10 code** | **ICD-10 Description** | **CHD diagnosis types (in order of decreasing complexity)** |  |
| --- | --- | --- | --- |
| Q234 | Hypoplastic left heart syndrome | Hypoplastic left heart syndrome (HLHS) |  |
| Q204 | Double inlet ventricle/Common ventricle/Tricuspid atresia /Single ventricle | Functionally univentricular heart (FUH) non-HLHS |  |
| Q226 | Hypoplastic right heart syndrome | Functionally univentricular heart (FUH) non-HLHS |  |
| Q224 | Congenital tricuspid stenosis atresia | Functionally univentricular heart (FUH) non-HLHS |  |
| Q205 | Discordant atrioventricular connection | Congenitally corrected transposition |  |
| Q206 | Isomerism of atrial appendages | Atrial isomerism with complex CHD |  |
| Q200 | Common arterial trunk / Persistent truncus arteriosus | Common arterial trunk |  |
| Q203 | Discordant ventriculoarterial connection / Dextro-transposition of great arteries | Transposition of great arteries (TGA, all types) |  |
| Q230 | Congenital stenosis of aortic valve | Aortic stenosis (valvar, sub-valvar) |  |
| Q244 | Congenital subaortic stenosis | Aortic stenosis (valvar, sub-valvar) |  |
| Q220 | Pulmonary valve atresia | Pulmonary atresia (intact septum or with VSD) |  |
| Q255 | Atresia of pulmonary artery | Pulmonary atresia (intact septum or with VSD) |  |
| Q213 | Tetralogy of Fallot / Ventricular septal defect with pulmonary stenosis or atresia, dextroposition of aorta and hypertrophy of right ventricle. | Tetralogy of Fallot (all types) |  |
| Q201 | Double outlet right ventricle / Taussig-Bing syndrome | Double outlet ventricle (Miscellaneous types) |  |
| Q202 | Double outlet left ventricle | Double outlet ventricle (Miscellaneous types) |  |
| Q212 | Atrioventricular septal defect | Atrioventricular septal defect (AVSD, all types) |  |
| Q262 | Total anomalous pulmonary venous connection | Totally anomalous pulmonary venous connection (TAPVC) |  |
| Q264 | Anomalous pulmonary venous connection, unspecified | Totally anomalous pulmonary venous connection (TAPVC) |  |
| Q252 | Atresia of aorta / Interrupted aortic arch | Aortic obstruction |  |
| Q251 | Coarctation of aorta | Aortic obstruction |  |
| Q253 | Stenosis of aorta / Supravalvular aortic stenosis | Aortic obstruction |  |
| Q254 | Other congenital malformations of aorta | Aortic arch malformations + vascular ring |  |
| Q225 | Ebstein’s anomaly | Tricuspid valve abnormality (including Ebstein's) |  |
| Q228 | Other congenital malformations of tricuspid valve | Tricuspid valve abnormality (including Ebstein's) |  |
| Q229 | Congenital malformation of tricuspid valve, unspecified | Tricuspid valve abnormality (including Ebstein's) |  |
| Q221 | Congenital pulmonary valve stenosis | Pulmonary stenosis |  |
| Q243 | Pulmonary infundibular stenosis | Pulmonary stenosis |  |
| Q233 | Congenital mitral insufficiency | Mitral - aortic valve diseases nonobstructive |  |
| Q238 | Other congenital malformations of aortic and mitral valves | Mitral - aortic valve diseases nonobstructive |  |
| Q239 | Congenital malformation of aortic and mitral valves, unspecified | Mitral - aortic valve diseases nonobstructive |  |
| Q231 | Congenital insufficiency of aortic valve / Congenital aortic insufficiency | Mitral - aortic valve diseases nonobstructive |  |
| Q222 | Congenital pulmonary valve insufficiency/regurgitation | Pulmonary other group - valve non obstructive or pulmonary artery diseases |  |
| Q223 | Other congenital malformations of pulmonary valve/Congenital malformation of pulmonary valve NOS | Pulmonary other group - valve non obstructive or pulmonary artery diseases |  |
| Q256 | Stenosis of pulmonary artery | Pulmonary other group - valve non obstructive or pulmonary artery diseases |  |
| Q257 | Other congenital malformations of pulmonary artery | Pulmonary other group - valve non obstructive or pulmonary artery diseases |  |
| Q210 | Ventricular septal defect/Roger's disease [Maladie de Roger]/Small VSD with no significant haemodynamic effects | Isolated ventricular septal defect (VSD) |  |
| Q232 | Congenital mitral stenosis / Congenital mitral atresia | Other ungrouped CHDs |  |
| Q218 | Other congenital malformations of cardiac septa | Other ungrouped CHDs |  |
| Q219 | Congenital malformation of cardiac septum, unspecified / Septal heart defect, NOS | Other ungrouped CHDs |  |
| Q263 | Partial anomalous pulmonary venous connection | Other ungrouped CHDs |  |
| Q242 | Cor triatriatum | Other ungrouped CHDs |  |
| Q214 | Aortopulmonary septal defect/Aortic septal defect/Aortopulmonary window/ | Other ungrouped CHDs |  |
| Q208 | Other congenital malformations of cardiac chambers and connections | Other ungrouped CHDs |  |
| Q209 | Congenital malformation of cardiac chambers and connections, unspecified | Other ungrouped CHDs |  |
| Q245 | Malformation of coronary vessels / Congenital coronary (artery) aneurysm | Other ungrouped CHDs |  |
| Q258 | Other congenital malformations of great arteries | Other ungrouped CHDs |  |
| Q259 | Congenital malformation of great arteries, unspecified | Other ungrouped CHDs |  |
| Q261 | Persistent left superior vena cava | Other ungrouped CHDs |  |
| Q240 | Dextrocardia | Other ungrouped CHDs |  |
| Q248 | Other specified congenital malformations of heart | Other ungrouped CHDs |  |
| Q249 | Congenital malformation of heart, unspecified | Other ungrouped CHDs |  |
| Q260 | Congenital stenosis of vena cava | Other ungrouped CHDs |  |
| Q265 | Anomalous portal venous connection | Other ungrouped CHDs |  |
| Q266 | Portal vein-hepatic artery fistula | Other ungrouped CHDs |  |
| Q268 | Other congenital malformations of great veins/Absence of vena cava (inferior) (superior)/Azygos continuation of inferior vena cava/Persistent left posterior cardinal vein/Scimitar syndrome | Other ungrouped CHDs |  |
| Q269 | Congenital malformation of great vein, unspecified /Anomaly of vena cava (inferior) (superior) | Other ungrouped CHDs |  |
| Q893 | Situs inversus | Other ungrouped CHDs |  |
| Q288 | Other specified congenital malformations of circulatory system/Congenital aneurysm, specified site/Congenital lymphatic abnormalities | Other ungrouped CHDs |  |
| Q289 | Congenital malformation of circulatory system, unspecified | Other ungrouped CHDs |  |
| Q278 | Other specified congenital malformations of peripheral vascular system/Absence, atresia of artery or vein Congenital | Other ungrouped CHDs |  |
| Q211 | Atrial septal defect | Atrial septal defect or patient ductus arteriosus (ASD or PDA) (excluded from this study) |  |
| Q250 | Patent ductus arteriosus/PDA/Patent ductus/Persistent ductus arteriosus | Atrial septal defect or patient ductus arteriosus (ASD or PDA) (excluded from this study) |  |
| Q241 | Laevocardia | Atrial septal defect or patient ductus arteriosus (ASD or PDA) (excluded from this study) |  |
| Q246 | Congenital heart block | Congenital arrhythmia (excluded from this study) |  |
| For cases with more than once code the highest ranked code and group applies. The mapping scheme was based on 3-digit ICD-10 codes, as over 80% of the codes did not use 4 digits in NCARDRS. | | | |

## Supplementary Table S2: Hierarchical Mapping from European Paediatric Cardiac Code (EPCC) to CHD diagnosis types.

For cases with more than once code the highest ranked code and group applies.

| **European Paediatric Cardiac Code (EPCC) and description** | **CHD diagnosis types (in order of decreasing complexity)** |
| --- | --- |
| 010109. Hypoplastic left heart syndrome | Hypoplastic left heart syndrome (HLHS) |
| 060201. Mitral atresia | Hypoplastic left heart syndrome (HLHS) |
| 060202. Mitral atresia with imperforate mitral valve | Hypoplastic left heart syndrome (HLHS) |
| 060226. Mitral atresia with absent valvar annulus (connection-junction) | Hypoplastic left heart syndrome (HLHS) |
| 091503. Aortic atresia | Hypoplastic left heart syndrome (HLHS) |
| 091506. Aortic valvar atresia | Hypoplastic left heart syndrome (HLHS) |
| 010114. Double inlet atrioventricular connection (double inlet ventricle) | Functionally univentricular heart (FUH) non-HLHS |
| 010122. Functionally univentricular heart | Functionally univentricular heart (FUH) non-HLHS |
| 010124. Double outlet right ventricle with intact ventricular septum | Functionally univentricular heart (FUH) non-HLHS |
| 010403. Double inlet right ventricle | Functionally univentricular heart (FUH) non-HLHS |
| 010404. Double inlet left ventricle | Functionally univentricular heart (FUH) non-HLHS |
| 010405. Double inlet to solitary ventricle of indeterminate morphology | Functionally univentricular heart (FUH) non-HLHS |
| 020303. Crisscross heart (twisted atrioventricular connections) | Functionally univentricular heart (FUH) non-HLHS |
| 020305. Solitary ventricle of indeterminate morphology | Functionally univentricular heart (FUH) non-HLHS |
| 060101. Tricuspid atresia | Functionally univentricular heart (FUH) non-HLHS |
| 060102. Tricuspid atresia with imperforate tricuspid valve | Functionally univentricular heart (FUH) non-HLHS |
| 060311. Congenital anomaly of right-sided atrioventricular valve in double inlet ventricle | Functionally univentricular heart (FUH) non-HLHS |
| 060411. Congenital anomaly of left-sided atrioventricular valve in double inlet ventricle | Functionally univentricular heart (FUH) non-HLHS |
| 060726. Atrioventricular septal defect (AVSD) with ventricular imbalance | Functionally univentricular heart (FUH) non-HLHS |
| 070841. Ventricular imbalance: dominant left ventricle + hypoplastic right ventricle | Functionally univentricular heart (FUH) non-HLHS |
| 070842. Ventricular imbalance: dominant right ventricle + hypoplastic left ventricle | Functionally univentricular heart (FUH) non-HLHS |
| 060126. Tricuspid atresia with absent valvar annulus (connection-junction) | Functionally univentricular heart (FUH) non-HLHS |
| 010119. Double outlet right ventricle with non-committed ventricular septal defect | Functionally univentricular heart (FUH) non-HLHS |
| 010103. Congenitally corrected transposition of great arteries (discordant atrioventricular & ventriculo-arterial connections) | Congenitally corrected transposition |
| 010401. Discordant atrioventricular connections | Congenitally corrected transposition |
| 030104. Right isomerism | Atrial isomerism with complex CHD |
| 030105. Left isomerism | Atrial isomerism with complex CHD |
| 090101. Common arterial trunk (truncus arteriosus) | Common arterial trunk |
| 090111. Common arterial trunk (truncus arteriosus) with aortic dominance and one pulmonary artery absent from trunk, isolated pulmonary artery | Common arterial trunk |
| 090112. Common arterial trunk (truncus arteriosus) with pulmonary dominance and aortic arch obstruction | Common arterial trunk |
| 090114. Common arterial trunk (truncus arteriosus) with aortic dominance and both pulmonary arteries arising from trunk | Common arterial trunk |
| 090115. Common arterial trunk (truncus arteriosus) with aortic dominance (no aortic arch obstruction) | Common arterial trunk |
| 090118. Common arterial trunk (truncus arteriosus) with pulmonary dominance and interrupted aortic arch | Common arterial trunk |
| 090119. Common arterial trunk (truncus arteriosus) with pulmonary dominance and aortic coarctation | Common arterial trunk |
| 090200. Truncal valvar abnormality | Common arterial trunk |
| 090201. Dysplasia of truncal valve | Common arterial trunk |
| 090203. Truncal valvar regurgitation | Common arterial trunk |
| 090218. Congenital truncal valvar stenosis | Common arterial trunk |
| 090219. Congenital truncal valvar regurgitation | Common arterial trunk |
| 010110. Transposition of the great arteries with concordant atrioventricular connections and ventricular septal defect | Transposition of great arteries (TGA, all types) |
| 010118. Double outlet right ventricle with subpulmonary ventricular septal defect, transposition type | Transposition of great arteries (TGA, all types) |
| 010501. Transposition of great arteries (discordant ventriculo-arterial connections) (TGA) | Transposition of great arteries (TGA, all types) |
| 010102. Transposition of the great arteries with concordant atrioventricular connections and intact ventricular septum | Transposition of great arteries (TGA, all types) |
| 091501. Congenital aortic valvar stenosis | Aortic stenosis (valvar, sub-valvar) |
| 091513. Aortic valvar stenosis | Aortic stenosis (valvar, sub-valvar) |
| 091517. Aortic 'annular' hypoplasia | Aortic stenosis (valvar, sub-valvar) |
| 091521. Unicuspid aortic valve | Aortic stenosis (valvar, sub-valvar) |
| 091592. Aortic stenosis | Aortic stenosis (valvar, sub-valvar) |
| 070900. Subaortic stenosis | Aortic stenosis (valvar, sub-valvar) |
| 070903. Subaortic stenosis due to fibromuscular shelf | Aortic stenosis (valvar, sub-valvar) |
| 070908. Left ventricular outflow tract obstruction due to atrioventricular valve | Aortic stenosis (valvar, sub-valvar) |
| 070916. Subaortic stenosis due to fibromuscular tunnel | Aortic stenosis (valvar, sub-valvar) |
| 070928. Congenital left ventricular outflow tract obstruction | Aortic stenosis (valvar, sub-valvar) |
| 070950. Congenital subaortic stenosis | Aortic stenosis (valvar, sub-valvar) |
| 070901. Left ventricular outflow tract obstruction | Aortic stenosis (valvar, sub-valvar) |
| 010107. Pulmonary atresia with intact ventricular septum | Pulmonary atresia (intact septum or with VSD) |
| 090511. Pulmonary atresia | Pulmonary atresia (intact septum or with VSD) |
| 090512. Pulmonary atresia: imperforate valve | Pulmonary atresia (intact septum or with VSD) |
| 090516. Congenital pulmonary atresia | Pulmonary atresia (intact septum or with VSD) |
| 010106. Pulmonary atresia + ventricular septal defect (VSD) (including Fallot type) | Tetralogy of Fallot (all types) |
| 010125. Pulmonary atresia + ventricular septal defect (VSD) + systemic-to-pulmonary collateral artery(ies) (MAPCA(s)) | Tetralogy of Fallot (all types) |
| 010126. Tetralogy of Fallot with pulmonary atresia | Tetralogy of Fallot (all types) |
| 010157. Tetralogy of Fallot with pulmonary atresia and systemic-to-pulmonary collateral artery(ies) (MAPCA(s)) | Tetralogy of Fallot (all types) |
| 090705. Absent or atretic pulmonary trunk (main pulmonary artery) | Tetralogy of Fallot (all types) |
| 090726. Solitary arterial trunk (absent intrapericardial pulmonary arteries) | Tetralogy of Fallot (all types) |
| 090801. Major systemic-to-pulmonary collateral artery(ies) (MAPCA(s)) | Tetralogy of Fallot (all types) |
| 090902. Right pulmonary artery from arterial duct | Tetralogy of Fallot (all types) |
| 090903. Right pulmonary artery from ascending aorta | Tetralogy of Fallot (all types) |
| 090904. Left pulmonary artery from arterial duct | Tetralogy of Fallot (all types) |
| 090905. Left pulmonary artery from ascending aorta | Tetralogy of Fallot (all types) |
| 090908. Pulmonary artery origin from ascending aorta (hemitruncus) | Tetralogy of Fallot (all types) |
| 090911. Pulmonary artery from arterial duct | Tetralogy of Fallot (all types) |
| 092025. Systemic-to-pulmonary collateral arter(ies) (MAPCA(s)) stenosis(es) | Tetralogy of Fallot (all types) |
| 090525. Tetralogy of Fallot with absent pulmonary valve syndrome | Tetralogy of Fallot (all types) |
| 010101. Tetralogy of Fallot | Tetralogy of Fallot (all types) |
| 010117. Double outlet right ventricle with subaortic or doubly committed ventricular septal defect and pulmonary stenosis, Fallot type | Tetralogy of Fallot (all types) |
| 010104. Double outlet right ventricle | Double outlet ventricle (Miscellaneous types) |
| 010140. Double outlet right ventricle with subaortic or doubly committed ventricular septal defect without pulmonary stenosis, ventricular septal defect type | Double outlet ventricle (Miscellaneous types) |
| 010503. Double outlet left ventricle | Double outlet ventricle (Miscellaneous types) |
| 010120. Atrioventricular septal defect and tetralogy of Fallot | Atrioventricular septal defect (AVSD, all types) |
| 050601. Common atrium (virtual absence of atrial septum) | Atrioventricular septal defect (AVSD, all types) |
| 050603. Common atrium with common atrioventricular junction | Atrioventricular septal defect (AVSD, all types) |
| 060501. Atypical atrioventricular valve in atrioventricular septal defect (AVSD) | Atrioventricular septal defect (AVSD, all types) |
| 060506. Atrioventricular septal defect (AVSD) atrioventricular valvar regurgitation | Atrioventricular septal defect (AVSD, all types) |
| 060514. Atypical common atrioventricular valve | Atrioventricular septal defect (AVSD, all types) |
| 060525. Double orifice of left ventricular component of common atrioventricular valve (left atrioventricular valve) | Atrioventricular septal defect (AVSD, all types) |
| 060560. Common atrioventricular valvar regurgitation | Atrioventricular septal defect (AVSD, all types) |
| 060571. Atypical right ventricular component of common atrioventricular valve (right atrioventricular valve) | Atrioventricular septal defect (AVSD, all types) |
| 060572. Atypical left ventricular component of common atrioventricular valve (left atrioventricular valve) | Atrioventricular septal defect (AVSD, all types) |
| 060598. Deficient mural leaflet of left ventricular component of common atrioventricular valve (left atrioventricular valve) | Atrioventricular septal defect (AVSD, all types) |
| 060600. Atrioventricular septal defect (AVSD) | Atrioventricular septal defect (AVSD, all types) |
| 060601. Atrioventricular septal defect with communication at the atrial level only (primum atrial septal defect) (partial atrioventricular canal defect) | Atrioventricular septal defect (AVSD, all types) |
| 060608. Atrioventricular septal defect with communication at the ventricular level only (atrioventricular canal defect with isolated ventricular communication) | Atrioventricular septal defect (AVSD, all types) |
| 060609. Atrioventricular septal defect (atrioventricular canal defect) with communication at atrial level and unrestrictive communication at ventricular level (Complete atrioventricular septal defect) | Atrioventricular septal defect (AVSD, all types) |
| 060610. Atrioventricular septal defect (atrioventricular canal defect) with communication at atrial level and restrictive communication at ventricular level (intermediate atrioventricular septal defect) | Atrioventricular septal defect (AVSD, all types) |
| 060705. Atrioventricular septal defect (AVSD) with ventricular imbalance with dominant right ventricle, hypoplastic left ventricle | Atrioventricular septal defect (AVSD, all types) |
| 060706. Atrioventricular septal defect (AVSD) with ventricular imbalance with dominant left ventricle, hypoplastic right ventricle | Atrioventricular septal defect (AVSD, all types) |
| 060727. Atrioventricular septal defect (AVSD) with balanced ventricles | Atrioventricular septal defect (AVSD, all types) |
| 060728. Common atrioventricular junction with spontaneous fibrous closure of atrioventricular septal defect (AVSD) | Atrioventricular septal defect (AVSD, all types) |
| 060736. Common atrioventricular valve with unbalanced commitment of valve to ventricles | Atrioventricular septal defect (AVSD, all types) |
| 060737. Common atrioventricular valve with unbalanced commitment of valve to right ventricle | Atrioventricular septal defect (AVSD, all types) |
| 060738. Common atrioventricular valve with unbalanced commitment of valve to left ventricle | Atrioventricular septal defect (AVSD, all types) |
| 040600. Total anomalous pulmonary venous connection of the supracardiac type | Totally anomalous pulmonary venous connection (TAPVC) |
| 040805. Totally anomalous pulmonary venous connection | Totally anomalous pulmonary venous connection (TAPVC) |
| 040806. Obstructed pulmonary venous pathway or connection | Totally anomalous pulmonary venous connection (TAPVC) |
| 040807. Anomalous pulmonary venous connection | Totally anomalous pulmonary venous connection (TAPVC) |
| 040810. Total anomalous pulmonary venous connection of the cardiac type | Totally anomalous pulmonary venous connection (TAPVC) |
| 040820. Total anomalous pulmonary venous connection of the infracardiac type | Totally anomalous pulmonary venous connection (TAPVC) |
| 040830. Total anomalous pulmonary venous connection of the mixed type | Totally anomalous pulmonary venous connection (TAPVC) |
| 092931. Interrupted aortic arch | Aortic obstruction |
| 092932. Interrupted aortic arch distal to subclavian artery, type A | Aortic obstruction |
| 092933. Interrupted aortic arch between subclavian & common carotid arteries, type B | Aortic obstruction |
| 092934. Interrupted aortic arch between carotid arteries, type C | Aortic obstruction |
| 010133. Left heart obstruction at multiple sites (including Shone syndrome) | Aortic obstruction |
| 091600. Supravalvar aortic stenosis | Aortic obstruction |
| 091618. Congenital supravalvar aortic stenosis | Aortic obstruction |
| 092901. Coarctation of aorta | Aortic obstruction |
| 092911. Aortic arch hypoplasia | Aortic obstruction |
| 092944. Descending thoracic or abdominal aortic coarctation | Aortic obstruction |
| 091602. Hypoplasia of ascending aorta | Aortic obstruction |
| 070934. Congenital anomaly of aorta and-or its branches | Aortic arch malformations + vascular ring |
| 093100. Vascular ring | Aortic arch malformations + vascular ring |
| 093134. Vascular ring of left aortic arch and right arterial duct or ligament | Aortic arch malformations + vascular ring |
| 093135. Vascular ring of right aortic arch and left arterial duct or ligament | Aortic arch malformations + vascular ring |
| 093140. Tracheo-esophageal compressive syndrome | Aortic arch malformations + vascular ring |
| 092809. Double aortic arch | Aortic arch malformations + vascular ring |
| 090906. Anomalous origin of left pulmonary artery from right pulmonary artery (pulmonary arterial sling) | Aortic arch malformations + vascular ring |
| 093023. Innominate artery compression syndrome | Aortic arch malformations + vascular ring |
| 060104. Tricuspid annular hypoplasia | Tricuspid valve abnormality (including Ebstein's) |
| 060107. Congenital tricuspid valvar stenosis | Tricuspid valve abnormality (including Ebstein's) |
| 060134. Ebstein malformation of tricuspid valve | Tricuspid valve abnormality (including Ebstein's) |
| 060192. Tricuspid stenosis | Tricuspid valve abnormality (including Ebstein's) |
| 060103. Dysplasia of tricuspid valve | Tricuspid valve abnormality (including Ebstein's) |
| 060125. Congenital tricuspid regurgitation | Tricuspid valve abnormality (including Ebstein's) |
| 060191. Tricuspid regurgitation | Tricuspid valve abnormality (including Ebstein's) |
| 060109. Straddling tricuspid valve | Tricuspid valve abnormality (including Ebstein's) |
| 070520. Congenital right ventricular outflow tract obstruction | Pulmonary stenosis |
| 070530. Subpulmonary stenosis | Pulmonary stenosis |
| 070532. Congenital subpulmonary stenosis | Pulmonary stenosis |
| 090501. Pulmonary valvar stenosis | Pulmonary stenosis |
| 090504. Congenital pulmonary valvar stenosis | Pulmonary stenosis |
| 090505. Pulmonary 'annular' hypoplasia | Pulmonary stenosis |
| 090592. Pulmonary stenosis | Pulmonary stenosis |
| 060203. Dysplasia of mitral valve | Mitral - aortic valve diseases nonobstructive |
| 060205. Overriding mitral valve | Mitral - aortic valve diseases nonobstructive |
| 060212. Mitral subvalvar apparatus abnormality | Mitral - aortic valve diseases nonobstructive |
| 060221. Congenital anomaly of mitral subvalvar apparatus | Mitral - aortic valve diseases nonobstructive |
| 060225. Congenital mitral regurgitation | Mitral - aortic valve diseases nonobstructive |
| 060235. Mitral valvar prolapse | Mitral - aortic valve diseases nonobstructive |
| 060236. True cleft of anterior mitral leaflet (without atrioventricular septal defect) | Mitral - aortic valve diseases nonobstructive |
| 060256. Parachute malformation of mitral valve | Mitral - aortic valve diseases nonobstructive |
| 060272. Congenital mitral valvar prolapse | Mitral - aortic valve diseases nonobstructive |
| 060291. Mitral regurgitation | Mitral - aortic valve diseases nonobstructive |
| 091507. Congenital aortic regurgitation | Mitral - aortic valve diseases nonobstructive |
| 091530. Aortic valvar prolapse | Mitral - aortic valve diseases nonobstructive |
| 091591. Aortic regurgitation | Mitral - aortic valve diseases nonobstructive |
| 060209. Straddling mitral valve | Mitral - aortic valve diseases nonobstructive |
| 091010. Discontinuous (non-confluent) right and left pulmonary arteries | Pulmonary other diseases group - valve non obstructive or pulmonary artery diseases |
| 091030. Congenitally discontinuous, non-confluent right and left pulmonary arteries | Pulmonary other diseases group - valve non obstructive or pulmonary artery diseases |
| 091075. Absent or atretic right pulmonary artery | Pulmonary other diseases group - valve non obstructive or pulmonary artery diseases |
| 091077. Absent or atretic left pulmonary artery | Pulmonary other diseases group - valve non obstructive or pulmonary artery diseases |
| 090711. Pulmonary trunk hypoplasia | Pulmonary other diseases group - valve non obstructive or pulmonary artery diseases |
| 090713. Supravalvar pulmonary trunk stenosis | Pulmonary other diseases group - valve non obstructive or pulmonary artery diseases |
| 090715. Congenital supravalvar pulmonary stenosis | Pulmonary other diseases group - valve non obstructive or pulmonary artery diseases |
| 090716. Congenital anomaly of pulmonary arterial tree | Pulmonary other diseases group - valve non obstructive or pulmonary artery diseases |
| 090719. Congenital pulmonary trunk (main pulmonary artery) anomaly | Pulmonary other diseases group - valve non obstructive or pulmonary artery diseases |
| 090720. Congenital pulmonary trunk hypoplasia | Pulmonary other diseases group - valve non obstructive or pulmonary artery diseases |
| 091001. Pulmonary arterial stenosis | Pulmonary other diseases group - valve non obstructive or pulmonary artery diseases |
| 091006. Peripheral pulmonary arterial stenoses or hypoplasia: at-beyond hilar bifurcation | Pulmonary other diseases group - valve non obstructive or pulmonary artery diseases |
| 091007. Central pulmonary arterial stenosis or hypoplasia: proximal to hilar bifurcation | Pulmonary other diseases group - valve non obstructive or pulmonary artery diseases |
| 091025. Right pulmonary arterial stenosis | Pulmonary other diseases group - valve non obstructive or pulmonary artery diseases |
| 091026. Left pulmonary arterial stenosis | Pulmonary other diseases group - valve non obstructive or pulmonary artery diseases |
| 091027. Congenital pulmonary arterial branch stenosis | Pulmonary other diseases group - valve non obstructive or pulmonary artery diseases |
| 091028. Congenital right pulmonary arterial stenosis | Pulmonary other diseases group - valve non obstructive or pulmonary artery diseases |
| 091029. Congenital left pulmonary arterial stenosis | Pulmonary other diseases group - valve non obstructive or pulmonary artery diseases |
| 091037. Congenital central pulmonary arterial stenosis or hypoplasia proximal to hilar bifurcation | Pulmonary other diseases group - valve non obstructive or pulmonary artery diseases |
| 091038. Congenital peripheral pulmonary arterial stenoses or hypoplasia at or beyond hilar bifurcation | Pulmonary other diseases group - valve non obstructive or pulmonary artery diseases |
| 091011. Pulmonary arterial hypoplasia | Pulmonary other diseases group - valve non obstructive or pulmonary artery diseases |
| 091071. Congenital pulmonary arterial hypoplasia | Pulmonary other diseases group - valve non obstructive or pulmonary artery diseases |
| 091072. Congenital right pulmonary artery hypoplasia | Pulmonary other diseases group - valve non obstructive or pulmonary artery diseases |
| 091073. Congenital left pulmonary artery hypoplasia | Pulmonary other diseases group - valve non obstructive or pulmonary artery diseases |
| 071000. Ventricular septal defect (VSD) | Isolated ventricular septal defect (VSD) |
| 071001. Perimembranous central ventricular septal defect (VSD) | Isolated ventricular septal defect (VSD) |
| 071002. Inlet perimembranous ventricular septal defect (VSD) without atrioventricular malalignment without a common atrioventricular junction | Isolated ventricular septal defect (VSD) |
| 071004. Outlet perimembranous ventricular septal defect (VSD) with anteriorly malaligned of outlet septum | Isolated ventricular septal defect (VSD) |
| 071012. Ventricular septal defect (VSD) with malaligned outlet septum | Isolated ventricular septal defect (VSD) |
| 071017. Outlet ventricular septal defect (VSD) with anteriorly malaligned outlet septum | Isolated ventricular septal defect (VSD) |
| 071018. Outlet ventricular septal defect (VSD) with posteriorly malaligned outlet septum | Isolated ventricular septal defect (VSD) |
| 071019. Outlet perimembranous ventricular septal defect (VSD) with posteriorly malaligned outlet septum | Isolated ventricular septal defect (VSD) |
| 071101. Trabecular muscular ventricular septal defect (VSD) | Isolated ventricular septal defect (VSD) |
| 071102. Inlet muscular ventricular septal defect (VSD) | Isolated ventricular septal defect (VSD) |
| 071103. Trabecular muscular ventricular septal defect (VSD) apical | Isolated ventricular septal defect (VSD) |
| 071104. Trabecular muscular ventricular septal defect (VSD) midseptal | Isolated ventricular septal defect (VSD) |
| 071105. Trabecular muscular ventricular septal defect (VSD)s multiple (Swiss cheese septum) | Isolated ventricular septal defect (VSD) |
| 071106. Outlet muscular ventricular septal defect (VSD) without malalignment | Isolated ventricular septal defect (VSD) |
| 071107. Trabecular muscular ventricular septal defect (VSD) anterosuperior | Isolated ventricular septal defect (VSD) |
| 071112. Trabecular muscular ventricular septal defect (VSD) postero-inferior | Isolated ventricular septal defect (VSD) |
| 071115. Outlet muscular ventricular septal defect (VSD) with anteriorly malaligned outlet septum | Isolated ventricular septal defect (VSD) |
| 071116. Outlet muscular ventricular septal defect (VSD) with posteriorly malaligned outlet septum | Isolated ventricular septal defect (VSD) |
| 071200. Outlet ventricular septal defect (VSD) | Isolated ventricular septal defect (VSD) |
| 071201. Doubly committed juxta-arterial ventricular septal defect (VSD) without malalignment | Isolated ventricular septal defect (VSD) |
| 071202. Doubly committed juxta-arterial ventricular septal defect (VSD) without malalignment and with muscular postero-inferior rim | Isolated ventricular septal defect (VSD) |
| 071203. Doubly committed juxta-arterial ventricular septal defect (VSD) without malalignment and with perimembranous extension | Isolated ventricular septal defect (VSD) |
| 071205. Doubly committed juxta-arterial ventricular septal defect (VSD) with anteriorly malaligned fibrous outlet septum and perimembranous extension | Isolated ventricular septal defect (VSD) |
| 071206. Doubly committed juxta-arterial ventricular septal defect (VSD) with posteriorly malaligned fibrous outlet septum and perimembranous extension | Isolated ventricular septal defect (VSD) |
| 071207. Doubly committed juxta-arterial ventricular septal defect (VSD) with anteriorly malaligned fibrous outlet septum and muscular postero-inferior rim | Isolated ventricular septal defect (VSD) |
| 071208. Doubly committed juxta-arterial ventricular septal defect (VSD) with posteriorly malaligned outlet septum and muscular postero-inferior rim | Isolated ventricular septal defect (VSD) |
| 071209. Outlet ventricular septal defect (VSD) without malalignment | Isolated ventricular septal defect (VSD) |
| 071212. Doubly committed juxta-arterial ventricular septal defect (VSD) with anteriorly malaligned fibrous outlet septum | Isolated ventricular septal defect (VSD) |
| 071213. Doubly committed juxta-arterial ventricular septal defect (VSD) with posteriorly malaligned fibrous outlet septum | Isolated ventricular septal defect (VSD) |
| 071402. Communication between left ventricle and right atrium (Gerbode defect) | Isolated ventricular septal defect (VSD) |
| 071405. Inlet ventricular septal defect (VSD) | Isolated ventricular septal defect (VSD) |
| 071406. Inlet perimembranous ventricular septal defect (VSD) with atrioventricular septal malalignment and without common atrioventricular junction | Isolated ventricular septal defect (VSD) |
| 071501. Ventricular septal defect(s): haemodynamically insignificant | Isolated ventricular septal defect (VSD) |
| 071504. Multiple ventricular septal defect (VSD)s | Isolated ventricular septal defect (VSD) |
| 071505. Single ventricular septal defect (VSD) | Isolated ventricular septal defect (VSD) |
| 060105. Overriding tricuspid valve | Other ungrouped CHDs |
| 090500. Pulmonary valvar abnormality | Other ungrouped CHDs |
| 091512. Eccentric opening of tricuspid aortic valve | Other ungrouped CHDs |
| 060204. Mitral annular hypoplasia | Other ungrouped CHDs |
| 060292. Mitral stenosis | Other ungrouped CHDs |
| 060293. Mitral valve stenosis | Other ungrouped CHDs |
| 050202. Supravalvar or intravalvar mitral ring | Other ungrouped CHDs |
| 060213. Mitral subvalvar stenosis | Other ungrouped CHDs |
| 060222. Congenital mitral subvalvar stenosis | Other ungrouped CHDs |
| 060207. Congenital mitral valvar stenosis | Other ungrouped CHDs |
| 010116. Partial anomalous pulmonary venous connection of Scimitar type | Other ungrouped CHDs |
| 050201. Divided left atrium (cor triatriatum) | Other ungrouped CHDs |
| 090401. Aortopulmonary window | Other ungrouped CHDs |
| 090407. Congenital aortopulmonary window | Other ungrouped CHDs |
| 040701. Partial anomalous pulmonary venous connection(s) | Other ungrouped CHDs |
| 020101. Extra-thoracic heart (ectopia cordis) | Other ungrouped CHDs |
| 040802. Congenital atresia of pulmonary vein(s) | Other ungrouped CHDs |
| 040804. Congenital anomaly of pulmonary vein(s) | Other ungrouped CHDs |
| 040831. Congenital pulmonary venous stenosis and-or hypoplasia | Other ungrouped CHDs |
| 040891. Pulmonary vein stenosis | Other ungrouped CHDs |
| 094101. Anomalous origin of coronary artery from pulmonary arterial tree | Other ungrouped CHDs |
| 094103. Anomalous origin of left coronary artery from pulmonary artery (ALCAPA) | Other ungrouped CHDs |
| 094221. Anomalous aortic origin of coronary artery with ventriculo-arterial concordance | Other ungrouped CHDs |
| 100353. Congenital heart tumour | Other ungrouped CHDs |
| 150503. Pulmonary venous obstruction | Other ungrouped CHDs |
| 060100. Tricuspid valvar abnormality | Other ungrouped CHDs |
| 060111. Congenital anomaly of tricuspid valve | Other ungrouped CHDs |
| 060200. Mitral valvar abnormality | Other ungrouped CHDs |
| 060211. Congenital anomaly of mitral valve | Other ungrouped CHDs |
| 010139. Cardiac abnormality | Other ungrouped CHDs |
| 010159. Structural developmental anomaly of heart and-or great vessels | Other ungrouped CHDs |
| 010160. Vascular abnormality | Other ungrouped CHDs |
| 010306. Abnormal atrial arrangement | Other ungrouped CHDs |
| 010309. Congenital anomaly of an atrioventricular and-or ventriculo-arterial connection(s) | Other ungrouped CHDs |
| 010510. Concordant VA connections with parallel great arteries (anatomically corrected malposition) | Other ungrouped CHDs |
| 020102. Dextrocardia: heart predominantly in right hemithorax | Other ungrouped CHDs |
| 020104. Midline heart (mesocardia) | Other ungrouped CHDs |
| 020109. Anomalous position-orientation of heart | Other ungrouped CHDs |
| 020301. Right hand pattern ventricular topology (D loop) | Other ungrouped CHDs |
| 020302. Left hand pattern ventricular topology (L loop) | Other ungrouped CHDs |
| 020400. Superior-inferior ('upstairs-downstairs') ventricular relationship | Other ungrouped CHDs |
| 020412. Abnormal ventricular relationships | Other ungrouped CHDs |
| 020612. Abnormal relationship of great arterial roots | Other ungrouped CHDs |
| 020704. Abnormal position or relationship of great vessels | Other ungrouped CHDs |
| 030103. Total mirror imagery (situs inversus) | Other ungrouped CHDs |
| 030113. Congenital anomaly of position and-or spatial relationships of thoraco-abdominal organs | Other ungrouped CHDs |
| 030223. Scimitar syndrome | Other ungrouped CHDs |
| 040007. Congenital anomaly of mediastinal vein(s) | Other ungrouped CHDs |
| 040100. Superior caval vein (SVC) abnormality | Other ungrouped CHDs |
| 040101. Left superior caval vein (SVC) to coronary sinus | Other ungrouped CHDs |
| 040102. Left superior caval vein (SVC) to left-sided atrium | Other ungrouped CHDs |
| 040105. Absent right superior caval vein (SVC) | Other ungrouped CHDs |
| 040109. Congenital anomaly of superior caval vein (SVC) | Other ungrouped CHDs |
| 040125. Left superior caval vein (SVC) | Other ungrouped CHDs |
| 040200. Hepatic vein abnormality | Other ungrouped CHDs |
| 040213. Anomalous hepatic venous connection to heart | Other ungrouped CHDs |
| 040300. Inferior caval vein (IVC) abnormality | Other ungrouped CHDs |
| 040308. Congenital anomaly of the inferior caval vein (IVC) | Other ungrouped CHDs |
| 040310. Interrupted inferior caval vein (IVC) with absent suprarenal segment and azygos continuation | Other ungrouped CHDs |
| 040400. Coronary sinus abnormality | Other ungrouped CHDs |
| 040405. Congenital anomaly of coronary sinus | Other ungrouped CHDs |
| 040413. Unroofed coronary sinus | Other ungrouped CHDs |
| 040414. Coronary sinus orifice atresia or stenosis | Other ungrouped CHDs |
| 040500. Congenital anomaly of systemic vein(s) | Other ungrouped CHDs |
| 040800. Pulmonary vein abnormality | Other ungrouped CHDs |
| 050100. Right atrial abnormality | Other ungrouped CHDs |
| 050106. Left-sided juxtaposition of the atrial appendages | Other ungrouped CHDs |
| 050112. Congenital giant right atrium | Other ungrouped CHDs |
| 050113. Congenital anomaly of right atrium | Other ungrouped CHDs |
| 050121. Divided right atrium (obstructive Eustachian valve) (cor triatriatum dexter) | Other ungrouped CHDs |
| 050200. Left atrial abnormality | Other ungrouped CHDs |
| 050204. Right-sided juxtaposition of the atrial appendages | Other ungrouped CHDs |
| 050211. Congenital anomaly of left atrium | Other ungrouped CHDs |
| 050300. Atrial septum abnormality | Other ungrouped CHDs |
| 050303. Aneurysm of the atrial septum | Other ungrouped CHDs |
| 050604. Restrictive interatrial communication or intact atrial septum when an interatrial shunt is physiologically necessary | Other ungrouped CHDs |
| 050701. Congenital anomaly of atrial septum | Other ungrouped CHDs |
| 070100. Right ventricular abnormality | Other ungrouped CHDs |
| 070106. Parchment right ventricle (including Uhl’s anomaly) | Other ungrouped CHDs |
| 070107. Congenital right ventricular anomaly | Other ungrouped CHDs |
| 070113. Right ventricular myocardial sinusoids | Other ungrouped CHDs |
| 070114. Right ventricular aneurysm | Other ungrouped CHDs |
| 070200. Right ventricular hypoplasia | Other ungrouped CHDs |
| 070301. Double chambered right ventricle | Other ungrouped CHDs |
| 070501. Right ventricular outflow tract obstruction | Other ungrouped CHDs |
| 070600. Left ventricular abnormality | Other ungrouped CHDs |
| 070607. Congenital left ventricular anomaly | Other ungrouped CHDs |
| 070612. Left ventricular myocardial sinusoids | Other ungrouped CHDs |
| 070613. Left ventricular aneurysm | Other ungrouped CHDs |
| 070619. Congenital left ventricular aneurysm or diverticulum | Other ungrouped CHDs |
| 070700. Left ventricular hypoplasia | Other ungrouped CHDs |
| 070931. Aortic abnormality | Other ungrouped CHDs |
| 071407. Restrictive interventricular communication when an interventricular shunt is physiologically necessary | Other ungrouped CHDs |
| 072000. Ventricular septal abnormality | Other ungrouped CHDs |
| 072004. Congenital anomaly of ventricular septum | Other ungrouped CHDs |
| 090428. Congenital anomaly of great arteries including arterial duct | Other ungrouped CHDs |
| 090429. Congenital anomaly of ventriculo-arterial valve(s) and-or adjacent regions | Other ungrouped CHDs |
| 090522. Congenital pulmonary regurgitation | Other ungrouped CHDs |
| 090524. Dysplasia of pulmonary valve | Other ungrouped CHDs |
| 090529. Congenital anomaly of pulmonary valve | Other ungrouped CHDs |
| 090532. Bicuspid pulmonary valve | Other ungrouped CHDs |
| 090700. Pulmonary trunk (MPA) abnormality | Other ungrouped CHDs |
| 090818. Systemic-to-pulmonary collateral artery(ies) | Other ungrouped CHDs |
| 091000. Pulmonary arterial abnormality | Other ungrouped CHDs |
| 091036. Congenital dilation of pulmonary arterial tree | Other ungrouped CHDs |
| 091041. Congenital pulmonary arterial branch anomaly | Other ungrouped CHDs |
| 091500. Aortic valvar abnormality | Other ungrouped CHDs |
| 091509. Dysplasia of aortic valve | Other ungrouped CHDs |
| 091519. Congenital anomaly of aortic valve | Other ungrouped CHDs |
| 091522. Bicuspid aortic valve | Other ungrouped CHDs |
| 091606. Congenital anomaly of ascending aorta | Other ungrouped CHDs |
| 091610. Ascending aorta abnormality | Other ungrouped CHDs |
| 091619. Congenital ascending aorta dilation or aneurysm | Other ungrouped CHDs |
| 091701. Aorto-ventricular tunnel | Other ungrouped CHDs |
| 091702. Aorto - left ventricular tunnel | Other ungrouped CHDs |
| 091801. Aneurysm of aortic sinus of Valsalva | Other ungrouped CHDs |
| 091901. Arteriovenous fistula (malformation) | Other ungrouped CHDs |
| 091905. Pulmonary arteriovenous fistula (malformation) | Other ungrouped CHDs |
| 092020. Distal systemic arterial abnormality | Other ungrouped CHDs |
| 092705. Congenital arterial duct (ductus arteriosus) anomaly | Other ungrouped CHDs |
| 092800. Aortic arch abnormality | Other ungrouped CHDs |
| 092806. Cervical aortic arch | Other ungrouped CHDs |
| 092810. Congenital anomaly of aortic arch | Other ungrouped CHDs |
| 092815. Right aortic arch | Other ungrouped CHDs |
| 092847. Congenital anomaly of descending thoracic and-or abdominal aorta | Other ungrouped CHDs |
| 092916. Descending-abdominal aorta hypoplasia (middle aortic syndrome) | Other ungrouped CHDs |
| 093000. Aortic arch branch abnormality | Other ungrouped CHDs |
| 093002. Aberrant origin right subclavian artery | Other ungrouped CHDs |
| 093004. Aberrant origin left subclavian artery | Other ungrouped CHDs |
| 093016. Isolation of an aortic arch branch | Other ungrouped CHDs |
| 093017. Congenital anomaly of aortic arch branch | Other ungrouped CHDs |
| 094200. Anomalous aortic origin or course of coronary artery | Other ungrouped CHDs |
| 094220. Anomalous aortic origin of coronary artery (AAOCA) | Other ungrouped CHDs |
| 094304. Anterior descending from right coronary artery across right ventricular outflow tract | Other ungrouped CHDs |
| 094305. Intramural proximal coronary arterial course | Other ungrouped CHDs |
| 094312. Myocardial bridging of coronary artery (intramyocardial coronary arterial course) | Other ungrouped CHDs |
| 094313. Single coronary supplying all of heart | Other ungrouped CHDs |
| 094318. Aberrant course of coronary artery: across right ventricular outflow tract | Other ungrouped CHDs |
| 094405. Congenital coronary arterial orifice stenosis | Other ungrouped CHDs |
| 094419. Congenital coronary arterial orifice atresia | Other ungrouped CHDs |
| 094501. Coronary fistula | Other ungrouped CHDs |
| 094510. Congenital coronary arterial fistula to right ventricle | Other ungrouped CHDs |
| 094511. Coronary fistulas within right ventricle ('sinusoidal') | Other ungrouped CHDs |
| 094516. Congenital coronary arterial fistula(s) | Other ungrouped CHDs |
| 094522. Congenital coronary arterial fistula to left ventricle | Other ungrouped CHDs |
| 094600. Coronary arterial abnormality | Other ungrouped CHDs |
| 094603. Congenital anomaly of coronary artery(ies) | Other ungrouped CHDs |
| 094606. Right ventricle dependent coronary circulation | Other ungrouped CHDs |
| 094614. Congenital coronary arterial aneurysm(s) | Other ungrouped CHDs |
| 094621. Left coronary artery from right aortic sinus with ventriculo-arterial concordance | Other ungrouped CHDs |
| 094626. Right coronary artery from left aortic sinus with ventriculo-arterial concordance | Other ungrouped CHDs |
| 050401. Interatrial communication ('ASD') | Atrial septal defect or patient ductus arteriosus (ASD or PDA) (excluded from this study) |
| 050402. Atrial septal defect (ASD) within oval fossa (secundum) | Atrial septal defect or patient ductus arteriosus (ASD or PDA) (excluded from this study) |
| 050403. Spontaneous closure of atrial septal defect (ASD) within oval fossa (secundum) | Atrial septal defect or patient ductus arteriosus (ASD or PDA) (excluded from this study) |
| 050500. Sinus venosus defect (ASD) | Atrial septal defect or patient ductus arteriosus (ASD or PDA) (excluded from this study) |
| 050503. Interatrial communication (ASD) through coronary sinus orifice | Atrial septal defect or patient ductus arteriosus (ASD or PDA) (excluded from this study) |
| 050602. Common atrium with separate atrioventricular junctions | Atrial septal defect or patient ductus arteriosus (ASD or PDA) (excluded from this study) |
| 092700. Arterial duct (ductus arteriosus) abnormality | Atrial septal defect or patient ductus arteriosus (ASD or PDA) (excluded from this study) |
| 092721. Patent arterial duct (PDA) | Atrial septal defect or patient ductus arteriosus (ASD or PDA) (excluded from this study) |
| This mapping was used for patients encountered in NCHDA only. For cases with more than once code the highest ranked code and group applies. | |

## Supplementary Table S3: Mapping from European Paediatric Cardiac Code (EPCC) to non-cardiac comorbidity groups.

| **European Paediatric Cardiac Code (EPCC) and description** | **Congenital non-cardiac comorbidity group** |
| --- | --- |
| 030102. Visceral heterotaxy (abnormal arrangement thoraco-abdominal organs) | Other anomalies / syndromes |
| 030109. Position or morphology of thoraco-abdominal organs abnormal | Other anomalies / syndromes |
| 030209. Lung anomaly | Respiratory |
| 030214. Functionally congenital single lung | Respiratory |
| 030305. Tracheobronchial anomaly | Respiratory |
| 030603. Intestines malrotated | Digestive system |
| 102049. Preprocedural gastrostomy present | Digestive system |
| 102304. Hereditary disorder potentially with associated heart disease | Other anomalies / syndromes |
| 140101. Chromosomal anomaly | Other anomalies / syndromes |
| 140102. Down Other anomalies / syndromes | Genetic |
| 140103. Trisomy Edwards' syndrome | Genetic |
| 140104. Trisomy Patau's syndrome | Genetic |
| 140105. 45XO: Turner’s syndrome | Genetic |
| 140120. Gene mutation or deletion | Genetic |
| 140121. 22q11 microdeletion | Genetic |
| 140200. Syndrome-association potentially with cardiac involvement | Other anomalies / syndromes |
| 140206. 22q11 microdeletion with full DiGeorge sequence (including immune dysfunction) | Genetic |
| 140210. Friedreich’s ataxia | Other anomalies / syndromes |
| 140217. Marfan syndrome | Other anomalies / syndromes |
| 140219. Noonan syndrome | Other anomalies / syndromes |
| 140221. Pompe’s disease: glycogen storage disease type IIa | Other anomalies / syndromes |
| 140228. Tuberous sclerosis | Other anomalies / syndromes |
| 140230. Williams syndrome (infantile hypercalcaemia) | Other anomalies / syndromes |
| 140232. Fetal rubella syndrome | Other anomalies / syndromes |
| 140234. Duchenne’s muscular dystrophy | Other anomalies / syndromes |
| 140258. Muscular dystrophy | Other anomalies / syndromes |
| 140262. Ehlers-Danlos syndrome | Other anomalies / syndromes |
| 140266. Alagille syndrome: arteriohepatic dysplasia | Other anomalies / syndromes |
| 140300. Noncardiac abnormality potentially with associated heart disease | Other anomalies / syndromes |
| 140304. Non-cardiothoracic-vascular abnormality | Other anomalies / syndromes |
| 140306. Cystic fibrosis | Other anomalies / syndromes |
| 140307. Congenital diaphragmatic hernia | Other anomalies / syndromes |
| 140308. Tracheo-oesophageal fistula | Digestive system |
| 140310. Omphalocoele (exomphalos) | Digestive system |
| 140311. Duodenal stenosis/atresia | Digestive system |
| 140321. Sickle cell disease | Other anomalies / syndromes |
| 140323. Renal abnormality | Urinary |
| 140328. Congenital coagulation disorder | Other anomalies / syndromes |
| 140329. Thoracic-mediastinal abnormality | Respiratory |
| 140333. Microcephaly | Nervous system |
| 140347. Choanal atresia | Respiratory |
| 140349. Tracheobronchial malacia | Respiratory |
| 140352. Hypothyroidism | Other anomalies / syndromes |
| 140391. Cerebral anomaly | Nervous system |
| 140392. Connective tissue disease | Other anomalies / syndromes |
| 140409. Kyphoscoliosis | Other anomalies / syndromes |
| 140412. Cleft lip or palate | Respiratory |
| 140415. Scoliosis | Other anomalies / syndromes |
| 140485. Loeys-Dietz Syndrome (transforming growth factor beta receptor (TGFBR) gene mutation) | Other anomalies / syndromes |
| 140490. Von Willebrand disease | Other anomalies / syndromes |
| 140540. Maternally derived fetal disease or syndrome potentially with associated heart disease | Other anomalies / syndromes |
| 140550. Major anomaly of gastrointestinal system | Digestive system |
| 140601. Multiple congenital malformations | Other anomalies / syndromes |
| 161001. Tracheal stenosis | Respiratory |
| 161009. Tracheal disease | Respiratory |

## Supplementary Table S4: Mappings between treating centers recorded in National Congenital Heart Disease Audit (NCHDA) and regions of England

| **Treating centre** | **Region (in England)** |
| --- | --- |
| Alder Hey Children's Hospital | North-West |
| Birmingham Children's Hospital | West Midlands |
| Bristol Royal Hospital for Children | South-West |
| Evelina London Children's Hospital | East of England/London and South-East/Thames Valley |
| Freeman hospital | Northern |
| Glenfield hospital | East Midlands and South Yorkshire |
| Great Ormond Street Hospital | East of England/London and South-East/Thames Valley |
| Leeds General Infirmary | Yorkshire and Humber |
| Royal Brompton hospital | East of England/London and South-East/Thames Valley |
| Southampton General Hospital | Wessex |

## Supplementary Table S5: Demographic and clinical characteristics of the cohort.

|  | **Whole cohort n=11,265** |
| --- | --- |
| **Demographic characteristics** | |
| **Gender** |  |
| Boy | 5,749 (51.0%) |
| Girl | 4,925 (43.7%) |
| Missing | 590 (5.2%) |
| **IMD score (level of relative deprivation)** |  |
| Quintile 1 (most deprived) | 3,185 (28.3%) |
| Quintile 2 | 2,618 (23.2%) |
| Quintile 3 | 2,135 (19.0%) |
| Quintile 4 | 1,771 (15.7%) |
| Quintile 5 (least deprived) | 1,505 (13.4%) |
| Missing | 51 (0.5%) |
| **Region (in England)** |  |
| London, South-East, East of England, and Thames Valley | 4,670 (41.5%) |
| West Midlands | 1,320 (11.7%) |
| North-West | 1,212 (10.8%) |
| East Midlands and South Yorkshire | 1,126 (10.0%) |
| South-West | 934 (8.3%) |
| Yorkshire and Humber | 833 (7.4%) |
| Northern | 572 (5.1%) |
| Wessex | 598 (5.3%) |
| **Clinical characteristics** | |
| **CHD diagnosis types (in order of decreasing complexity)** |  |
| Hypoplastic left heart syndrome (HLHS) | 556 (4.9%) |
| Functionally univentricular heart (FUH) non-HLHS | 303 (2.7%) |
| Congenitally corrected transposition | 71 (0.6%) |
| Atrial isomerism with complex CHD | 171 (1.5%) |
| Common arterial trunk | 142 (1.3%) |
| Transposition of great arteries (all types) | 533 (4.7%) |
| Aortic stenosis (valvar, subvalvar) | 326 (2.9%) |
| Pulmonary atresia (intact septum or with VSD) | 273 (2.4%) |
| Tetralogy of Fallot (all types) | 794 (7.0%) |
| Double outlet ventricle (Misc) | 278 (2.5%) |
| Atrioventricular septal defect (all types) | 907 (8.1%) |
| Totally anomalous pulmonary venous connection (TAPVC) | 148 (1.3%) |
| Aortic obstruction | 848 (7.5%) |
| Aortic arch malformations + vascular ring | 842 (7.5%) |
| Tricuspid valve abnormality (including Ebstein's) | 315 (2.8%) |
| Pulmonary stenosis | 507 (4.5%) |
| Mitral - aortic valve diseases nonobstructive | 260 (2.3%) |
| Pulmonary other diseases (valve non obstructive or pulmonary artery diseases) | 210 (1.9%) |
| Isolated ventricular septal defect (VSD) | 2,981 (26.5%) |
| Other ungrouped CHDs | 800 (7.1%) |
| **Comorbidity and subtypes** |  |
| Without comorbidity | 7,282 (64.6%) |
| With comorbidity (any type) | 3,983 (35.4%) |
| Genetic | 2,040 (18.1%) |
| Digestive system | 700 (6.2%) |
| Urinary | 496 (4.4%) |
| Limb | 386 (3.4%) |
| Nervous system | 398 (3.5%) |
| Oro-facial clefts/Eye/Ear, face and neck | 341 (3.0%) |
| Respiratory | 466 (4.1%) |
| Abdominal wall defects | 184 (1.6%) |
| Genital | 166 (1.5%) |
| Other anomalies / syndromes | 972 (8.6%) |
| **Antenatal detection** | 7,173 (63.7%) |
| **Pre-term birth (<37 weeks' gestation)*** | 2,339 (25.4%) |
| * based on the presence in n=9,204 live birth. | |

## Supplementary Table S6: The number and proportions of antenatal detection based on demographic and clinical characteristics

|  | **Number of cases** | **Number of cases with antenatal detection** | **Antenatal detection rate with 95% confidence interval** | **p-value**^†^ |
| --- | --- | --- | --- | --- |
| **Whole cohort** | 11,265 | 7,173 | 63.7% (62.8%-64.6%) |  |
| **Demographic characteristics** | | | | |
| **Gender** | | |  | p=0.52 |
| Boy | 5,749 | 3,529 | 61.4% (60.1%-62.6%) |  |
| Girl | 4,925 | 3,054 | 62.0% (60.6%-63.4%) |  |
| Missing data | 590 | 590 | 99.8% (99.1%-100.0%) |  |
| **IMD score (level of relative deprivation)** | | |  | p=0.04 |
| Quintile 1 (most deprived) | 3,185 | 1,977 | 62.1% (60.4%-63.8%) |  |
| Quintile 2 | 2,618 | 1,684 | 64.3% (62.5%-66.2%) |  |
| Quintile 3 | 2,135 | 1,383 | 64.8% (62.7%-66.8%) |  |
| Quintile 4 | 1,771 | 1,108 | 62.6% (60.3%-64.8%) |  |
| Quintile 5 (least deprived) | 1,505 | 995 | 66.1% (63.7%-68.5%) |  |
| Missing data | 51 | 26 | 51.0% (36.6%-65.2%) |  |
| **Region (in England)** | | |  | p<0.001 |
| East of England/London and South-East/Thames Valley | 4,670 | 3,220 | 69.0% (67.6%-70.3%) |  |
| West Midlands | 1,320 | 768 | 58.2% (55.5%-60.9%) |  |
| North-West | 1,212 | 712 | 58.7% (55.9%-61.5%) |  |
| East Midlands and South Yorkshire | 1,126 | 762 | 67.7% (64.9%-70.4%) |  |
| South-West | 934 | 605 | 64.8% (61.6%-67.8%) |  |
| Yorkshire and Humber | 833 | 463 | 55.6% (52.1%-59.0%) |  |
| Northern | 572 | 308 | 53.8% (49.7%-58.0%) |  |
| Wessex | 598 | 335 | 56.0% (51.9%-60.0%) |  |
| **Clinical characteristics** | | | | |
| **CHD diagnosis types (results not shown here; please refer to Table 1 in the main text)** | | |  |  |
| **Congenital non-cardiac comorbidity** | | |  | p<0.001^‡^ |
| Without comorbidity (any type) | 7,282 | 4,152 | 57.0% (55.9%-58.2%) |  |
| With comorbidity (any type) | 3,983 | 3,021 | 75.8% (74.5%-77.2%) |  |
| Genetic | 2,040 | 1,612 | 79.0% (77.2%-80.8%) |  |
| Digestive system | 700 | 534) | 76.3% (73.0%-79.4%) |  |
| Urinary | 496 | 416 | 83.9% (80.3%-87.0%) |  |
| Limb | 386 | 325 | 84.2% (80.2%-87.7%) |  |
| Nervous system | 398 | 346 | 86.9% (83.2%-90.1%) |  |
| Oro-facial clefts/Eye/Ear, face and neck | 341 | 276 | 80.9% (76.4%-85.0%) |  |
| Respiratory | 466 | 334 | 71.7% (67.3%-75.7%) |  |
| Abdominal wall defects | 184 | 184 | 100 % (98.0%-100%) |  |
| Genital | 166 | 110 | 66.3% (58.5%-73.4%) |  |
| Other anomalies / syndromes | 972 | 684 | 70.4% (67.4%-73.2%) |  |
| **Preterm birth (<37 weeks' gestation)** | | | | p<0.001 |
| Live-born babies with preterm birth | 2,339 | 1,170 | 50.0% (48.0%-52.1%) |  |
| Live-born babies with full-term birth | 6,864 | 3,967 | 57.8% (56.6%-59.0%) |  |
| ^†^P-value for the chi-square test comparing antenatal detection among levels of demographic and clinical characteristics. Missing data were excluded from the analysis.  ^‡^Test for comparing pathway journeys among cases with any type of comorbidity vs. those without comorbidity. | | | | |

## Supplementary Table S7: **Number and proportion of structural CHD cases with comorbidities (any types) in different health care journeys by CHD types (**data for Figure 2).

|  | **All cases** | | **Fetuses with termination of pregnancy** | | **Stillborn or miscarried babies** | | **Live-born babies with cardiac intervention(s) in infancy** | | **Live-born babies with no cardiac intervention in infancy** | |
| --- | --- | --- | --- | --- | --- | --- | --- | --- | --- | --- |
|  | **Number of cases** | **Presence of comorbidity: n (%)** | **Number of cases** | **Presence of comorbidity: n (%)** | **Number of cases** | **Presence of comorbidity: n (%)** | **Number of cases** | **Presence of comorbidity: n (%)** | **Number of cases** | **Presence of comorbidity: n (%)** |
| **The whole cohort** | 11,265 | 3,983 (35.4%) | 1,766 | 941 (53.3%) | 295 | 192 (65.1%) | 4,538 | 1,389 (30.6%) | 4,666 | 1,461 (31.3%) |
| Hypoplastic left heart syndrome (HLHS) | 556 | 134 (24.1%) | 284 | 62 (21.8%) | 18 | 10 (55.6%) | 76 | 26 (34.2%) | 178 | 36 (20.2%) |
| Functionally univentricular heart (FUH) non-HLHS | 303 | 70 (23.1%) | 118 | 20 (16.9%) | 6 | * | 27 | 11 (40.7%) | 152 | 35 (23.0%) |
| Congenitally corrected transposition | 71 | 18 (25.4%) | 16 | * | * | * | 21 | * | 33 | 11 (33.3%) |
| Atrial isomerism with complex CHD | 171 | 104 (60.8%) | 48 | 22 (45.8%) | * | * | 74 | 47 (63.5%) | 44 | 34 (77.3%) |
| Common arterial trunk | 142 | 61 (43.0%) | 55 | 27 (49.1%) | * | * | 12 | * | 70 | 26 (37.1%) |
| Transposition of great arteries (TGA, all types) | 533 | 61 (11.4%) | 53 | 12 (22.6%) | 9 | * | 15 | * | 456 | 43 (9.4%) |
| Aortic stenosis (valvar, subvalvar) | 326 | 72 (22.1%) | 27 | 13 (48.1%) | * | * | 84 | 18 (21.4%) | 212 | 39 (18.4%) |
| Pulmonary atresia (intact septum or with VSD) | 273 | 99 (36.3%) | 93 | 33 (35.5%) | 13 | * | 27 | 14 (51.9%) | 140 | 47 (33.6%) |
| Tetralogy of Fallot (all types) | 794 | 313 (39.4%) | 126 | 70 (55.6%) | 21 | 11 (52.4%) | 79 | 41 (51.9%) | 568 | 191 (33.6%) |
| Double outlet ventricle (Misc types) | 278 | 123 (44.2%) | 107 | 44 (41.1%) | 15 | 12 (80.0%) | 33 | 20 (60.6%) | 123 | 47 (38.2%) |
| Atrioventricular septal defect (AVSD, all types) | 907 | 700 (77.2%) | 308 | 251 (81.5%) | 41 | 32 (78.0%) | 173 | 113 (65.3%) | 385 | 304 (79.0%) |
| Totally anomalous pulmonary venous connection (TAPVC) | 148 | 20 (13.5%) | 0 | N/A | 0 | N/A | 18 | 7 (38.9%) | 130 | 13 (10.0%) |
| Aortic obstruction | 848 | 265 (31.2%) | 76 | 44 (57.9%) | 29 | 22 (75.9%) | 148 | 51 (34.5%) | 595 | 148 (24.9%) |
| Aortic arch malformations + vascular ring | 842 | 291 (34.6%) | 71 | 53 (74.6%) | 24 | 20 (83.3%) | 531 | 137 (25.8%) | 216 | 81 (37.5%) |
| Tricuspid valve abnormality (including Ebstein's) | 315 | 93 (29.5%) | 46 | 20 (43.5%) | 13 | * | 187 | 51 (27.3%) | 69 | 17 (24.6%) |
| Pulmonary stenosis | 507 | 97 (19.1%) | 16 | 6 (37.5%) | * | * | 196 | 39 (19.9%) | 291 | 51 (17.5%) |
| Mitral - aortic valve diseases nonobstructive | 260 | 80 (30.8%) | 6 | 6 (100.0%) | * | * | 177 | 51 (28.8%) | 76 | 22 (28.9%) |
| Pulmonary other group - valve non obstructive or pulmonary artery diseases | 210 | 92 (43.8%) | 13 | 8 (61.5%) | * | * | 156 | 64 (41.0%) | 38 | 18 (47.4%) |
| Isolated ventricular septal defect (VSD) | 2,981 | 959 (32.2%) | 179 | 149 (83.2%) | 52 | 35 (67.3%) | 2,031 | 527 (25.9%) | 719 | 248 (34.5%) |
| Other ungrouped CHDs | 800 | 331 (41.4%) | 124 | 98 (79.0%) | 32 | 20 (62.5%) | 473 | 163 (34.5%) | 171 | 50 (29.2%) |
| *Sample size 1-5 (number suppressed) | | | | | | | | | | |

## Supplementary Table S8: The distribution of patient healthcare journeys categorized by demographic and clinical characteristics.

|  | **Number of cases** | **Number of cases that followed each distinct healthcare journey, along with the corresponding rate and 95% confidence interval** | | | | **p-value**^†^ |
| --- | --- | --- | --- | --- | --- | --- |
|  |  | **Foetuses with termination of pregnancy** | **Stillborn or miscarried babies** | **Live-born babies who had no cardiac intervention in infancy** | **Live-born babies who underwent cardiac intervention(s) in infancy** |  |
| **Whole cohort** | 11,265 | 1,766; 15.7% (14.7%-16.7%) | 295; 2.6% (1.6%-3.6%) | 4,538; 40.3% (39.3%-41.3%) | 4,666; 41.4% (40.4%-42.4%) |  |
| **Demographic characteristics** | | | | | | |
| **Gender** | | | | | |  |
| Boy | 5,749 | 628; 10.9% (9.5%-12.3%) | 132; 2.3% (0.9%-3.7%) | 2,289; 39.8% (38.4%-41.2%) | 2,700; 47.0% (45.6%-48.4%) | p<0.001 |
| Girl | 4,925 | 581; 11.8% (10.3%-13.3%) | 134; 2.7% (1.2%-4.2%) | 2,245; 45.6% (44.1%-47.1%) | 1,965; 39.9% (38.4%-41.4%) |  |
| Missing | 590 | 557; 94.2% (92.6%-96.0%) | 29; 4.9% (3.2%-6.7%) | * | * |  |
| **IMD score (level of relative deprivation)** | | | | | | p<0.001 |
| Quintile 1 (most deprived) | 3,185 | 369; 11.6% (9.7%-13.5%) | 115; 3.6% (1.8%-5.5%) | 1,340; 42.1% (40.2%-44.0%) | 1,361; 42.7% (40.9%-44.6%) |  |
| Quintile 2 | 2,618 | 393; 15.0% (12.9%-17.1%) | 85; 3.2% (1.2%-5.3%) | 1,047; 40.0% (37.9%-42.1%) | 1,093; 41.7% (39.7%-43.8%) |  |
| Quintile 3 | 2,135 | 382; 17.9% (15.6%-20.2%) | 39; 1.8% (0.0%-4.2%) | 841; 39.4% (37.1%-41.7%) | 873; 40.9% (38.6%-43.2%) |  |
| Quintile 4 | 1,771 | 313; 17.7% (15.2%-20.3%) | 30; 1.7% (0.0%-4.3%) | 711; 40.1% (37.7%-42.7%) | 717; 40.5% (38.0%-43.1%) |  |
| Quintile 5 (least deprived) | 1,505 | 305; 20.3% (17.5%-23.1%) | 25; 1.7% (0.0%-4.5%) | 585; 38.9% (36.1%-41.7%) | 590; 39.2% (36.5%-42.0%) |  |
| Missing | 51 | * | * | 14; 27.5% (15.7%-41.5%) | 32; 62.7% (51.0%-76.8%) |  |
| **Region (in England)** | | | | | | p<0.001 |
| London, South-East, East of England, and Thames Valley | 4,670 | 826; 17.7% (16.1%-19.3%) | 111; 2.4% (0.8%-3.9%) | 1,973; 42.2% (40.7%-43.8%) | 1,760; 37.7% (36.1%-39.3%) |  |
| West Midlands | 1,320 | 188; 14.2% (11.4%-17.2%) | 55; 4.2% (1.3%-7.1%) | 514; 38.9% (36.1%-41.9%) | 563; 42.7% (39.8%-45.6%) |  |
| North-West | 1,212 | 151; 12.5% (9.5%-15.5%) | 37; 3.1% (0.1%-6.1%) | 442; 36.5% (33.5%-39.5%) | 582; 48.0% (45.0%-51.1%) |  |
| East Midlands and South Yorkshire | 1,126 | 156; 13.9% (10.7%-17.0%) | 21; 1.9% (0.0%-5.0%) | 445; 39.5% (36.4%-42.7%) | 504; 44.8% (41.7%-47.9%) |  |
| South-West | 934 | 146; 15.6% (12.3%-19.2%) | 18; 1.9% (0.0%-5.4%) | 457; 48.9% (45.6%-52.4%) | 313; 33.5% (30.2%-37.0%) |  |
| Yorkshire and Humber | 833 | 110; 13.2% (9.6%-16.8%) | 24; 2.9% (0.0%-6.5%) | 305; 36.6% (33.0%-40.3%) | 394; 47.3% (43.7%-50.9%) |  |
| Northern | 572 | 77; 13.5% (9.3%-18.0%) | 15; 2.6% (0.0%-7.1%) | 206; 36.0% (31.8%-40.5%) | 274; 47.9% (43.7%-52.4%) |  |
| Wessex | 598 | 112; 18.7% (14.5%-23.1%) | 14; 2.3% (0.0%-6.8%) | 196; 32.8% (28.6%-37.2%) | 276; 46.2% (42.0%-50.6%) |  |
| **Clinical characteristics** | | | | | | |
| **CHD diagnosis types (results not shown here; please refer to Table 2 in the main text)** | | | | | |  |
| **Congenital non-cardiac comorbidity** |  |  |  |  |  | p<0.001^‡^ |
| Without comorbidity | 7,282 | 825; 11.3% (10.1%-12.6%) | 103; 1.4% (0.2%-2.7%) | 3,149; 43.2% (42.0%-44.5%) | 3,205; 44.0% (42.8%-45.3%) |  |
| With comorbidity (any type) | 3,983 | 941; 23.6% (21.9%-25.4%) | 192; 4.8% (3.1%-6.6%) | 1,389; 34.9% (33.2%-36.6%) | 1,461; 36.7% (35.0%-38.4%) |  |
| Genetic | 2040 | 611; 30.0% (27.6%-32.4%) | 127; 6.2% (3.9%-8.6%) | 574; 28.1% (25.8%-30.5%) | 728; 35.7% (33.3%-38.1%) |  |
| Digestive system | 700 | 119; 17.0% (13.1%-21.0%) | 23; 3.3% (0.0%-7.3%) | 340; 48.6% (44.7%-52.6%) | 218; 31.1% (27.3%-35.2%) |  |
| Urinary | 496 | 138; 27.8% (23.2%-32.8%) | 21; 4.2% (0.0%-9.2%) | 161; 32.5% (27.8%-37.4%) | 176; 35.5% (30.8%-40.4%) |  |
| Limb | 386 | 134; 34.7% (29.5%-40.2%) | 29; 7.5% (2.3%-13.0%) | 153; 39.6% (34.5%-45.1%) | 70; 18.1% (13.0%-23.6%) |  |
| Nervous system | 398 | 152; 38.2% (32.9%-43.5%) | 21; 5.3% (0.0%-10.6%) | 137; 34.4% (29.1%-39.7%) | 88; 22.1% (16.8%-27.4%) |  |
| Oro-facial clefts/Eye/Ear, face and neck | 341 | 95; 27.9% (22.3%-33.6%) | 20; 5.9% (0.3%-11.6%) | 149; 43.7% (38.1%-49.4%) | 77; 22.6% (17.0%-28.3%) |  |
| Respiratory | 466 | 44; 9.4% (5.4%-13.8%) | 5; 1.1% (0.0%-5.4%) | 100; 21.5% (17.4%-25.8%) | 317; 68.0% (63.9%-72.4%) |  |
| Abdominal wall defects | 184 | 97; 52.7% (45.7%-60.3%) | 20; 10.9% (3.8%-18.5%) | 49; 26.6% (19.6%-34.2%) | 18; 9.8% (2.7%-17.4%) |  |
| Genital | 166 | 31; 18.7% (10.8%-26.8%) | * | 80; 48.2% (40.4%-56.3%) | 51; 30.7% (22.9%-38.8%) |  |
| Other anomalies / syndromes | 972 | 107; 11.0% (7.9%-14.3%) | 13; 1.3% (0.0%-4.6%) | 273; 28.1% (25.0%-31.4%) | 579; 59.6% (56.5%-62.8%) |  |
| **Preterm birth (<37 weeks' gestation) (live birth only)** | | | | | | p<0.001 |
| Live-born babies with preterm birth | 2,339 | N/A | N/A | 1,454; 62.2% (60.2%-64.1%) | 885; 37.8% (35.9%-39.8%) |  |
| Live-born babies with full term birth | 6,864 | N/A | N/A | 3,083; 44.9% (43.7%-46.1%) | 3,781; 55.1% (53.9%-56.3%) |  |
| *Sample size 1-5 (number suppressed)  ^†^P-value for the chi-square test comparing pathway journeys among levels of demographic and clinical characteristics. Missing data were excluded from the analysis.  ^‡^ Test for comparing pathway journeys among cases with any type of comorbidity vs. those without comorbidity. | | | | | | |

## Supplementary Table S9: Infant mortality rates for live-born babies with CHD by patient journey and diagnosis types, with a breakdown by the presence of non-cardiac comorbidities (data of Figure 3).

|  | **Live birth with no cardiac intervention in infancy** | | | | **live birth with cardiac intervention(s) in infancy** | | | |
| --- | --- | --- | --- | --- | --- | --- | --- | --- |
|  | **Number of patients** | **Total infant death** | **Infant death with comorbidity** | **Infant death without comorbidity** | **Number of patients** | **Total infant death** | **Infant death with comorbidity** | **Infant death without comorbidity** |
| **All patients** | 4,520 | 602 (13.3%) | 362 (8.0%) | 240 (5.3%) | 4,620 | 243 (5.3%) | 118 (2.6%) | 125 (2.7%) |
| **CHD diagnosis types (in order of decreasing complexity)** | | | | | | | | |
| Hypoplastic left heart syndrome (HLHS) | 76 | 71 (93.4%) | 26 (34.2%) | 45 (59.2%) | 178 | 48 (27.0%) | 14 (7.9%) | 34 (19.1%) |
| Functionally univentricular heart (FUH) non-HLHS | 27 | 16 (59.3%) | 9 (33.3%) | 7 (25.9%) | 152 | 11 (7.2%) | * | * |
| Congenitally corrected transposition | 21 | * | 0 (0%) | * | 33 | * | * | * |
| Atrial isomerism with complex CHD | 74 | 17 (23.0%) | 11 (14.9%) | 6 (8.1%) | 44 | 6 (13.6%) | * | * |
| Common arterial trunk | 12 | 11 (91.7%) | * | * | 70 | 6 (8.6%) | * | * |
| Transposition of great arteries (TGA, all types) | 15 | 9 (60.0%) | * | * | 452 | 26 (5.8%) | * | * |
| Aortic stenosis (valvar, subvalvar) | 84 | 13 (15.5%) | * | * | 209 | 11 (5.3%) | * | * |
| Pulmonary atresia (intact septum or with VSD) | 27 | 14 (51.9%) | 8 (29.6%) | 6 (22.2%) | 140 | 11 (7.9%) | * | * |
| Tetralogy of Fallot (all types) | 78 | 30 (38.5%) | 22 (28.2%) | 8 (10.3%) | 564 | 17 (3.0%) | 12 (2.1%) | * |
| Double outlet ventricle (Misc types) | 33 | 20 (60.6%) | 14 (42.4%) | 6 (18.2%) | 123 | 11 (8.9%) | 6 (4.9%) | * |
| Atrioventricular septal defect (AVSD, all types) | 173 | 52 (30.1%) | 40 (23.1%) | 12 (6.9%) | 380 | 37 (9.7%) | 30 (7.9%) | 7 (1.8%) |
| Totally anomalous pulmonary venous connection (TAPVC) | 18 | 13 (72.2%) | 6 (33.3%) | 7 (38.9%) | 130 | * | * | * |
| Aortic obstruction | 148 | 34 (23.0%) | 17 (11.5%) | 17 (11.5%) | 588 | 16 (2.7%) | 12 (2.0%) | * |
| Aortic arch malformations + vascular ring | 526 | 33 (6.3%) | 21 (4.0%) | 12 (2.3%) | 215 | 7 (3.3%) | * | * |
| Tricuspid valve abnormality (including Ebstein's) | 185 | 20 (10.8%) | * | 15 (8.1%) | 69 | * | 0 (0%) | * |
| Pulmonary stenosis | 195 | * | * | * | 286 | * | 0 (0%) | * |
| Mitral - aortic valve diseases nonobstructive | 177 | 27 (15.3%) | 15 (8.5%) | 12 (6.8%) | 74 | * | * | * |
| Pulmonary other group - valve non obstructive or pulmonary artery diseases | 156 | 12 (7.7%) | * | * | 38 | 0 (0%) | 0 (0%) | 0 (0%) |
| Isolated ventricular septal defect (VSD) | 2023 | 142 (7.0%) | 112 (5.5%) | 30 (1.5%) | 706 | 11 (1.6%) | * | * |
| Other ungrouped CHDs | 472 | 63 (13.3%) | 35 (7.4%) | 28 (5.9%) | 169 | 11 (6.5%) | * | * |
| n=64 patients (non-intervened: 18; and intervened: 46) who had missing data in ONS or censored before the age of one year were removed from the analysis.  *Sample size 1-5 (number suppressed). Counts are also not shown if the suppressed numbers could be recalculated from them. | | | | | | | | |

## Supplementary Table S10: Healthcare journeys in live-born babies categorized by demographic and clinical characteristics.

|  | **Number of live birth** | **Number of cases that followed each distinct healthcare journey, along with the corresponding rate and 95% confidence interval** | | **p-value**^†^ |
| --- | --- | --- | --- | --- |
|  |  | **Live-born babies who had no cardiac intervention in infancy** | **Live-born babies who underwent cardiac intervention in infancy** |  |
| **Whole cohort** | 9,204 | 4,538; 49.3% (48.3%-50.3%) | 4,666; 50.7% (49.7%-51.7%) |  |
| **Demographic characteristics** | | | | |
| **Gender** | | | |  |
| Boy | 4,989 | 2,289; 45.9% (44.5%-47.3%) | 2,700; 54.1% (52.7%-55.5%) | p<0.001 |
| Girl | 4,210 | 2,245; 53.3% (51.8%-54.8%) | 1,965; 46.7% (45.2%-48.2%) |  |
| Missing | * | * | * |  |
| **IMD score (level of relative deprivation)** | | | | p=0.98 |
| Quintile 1 (most deprived) | 2,701 | 1,340; 49.6% (47.7%-51.5%) | 1,361; 50.4% (48.5%-52.3%) |  |
| Quintile 2 | 2,140 | 1,047; 48.9% (46.8%-51.1%) | 1,093; 51.1% (48.9%-53.2%) |  |
| Quintile 3 | 1,714 | 841; 49.1% (46.7%-51.5%) | 873; 50.9% (48.5%-53.3%) |  |
| Quintile 4 | 1,428 | 711; 49.8% (47.2%-52.4%) | 717; 50.2% (47.6%-52.8%) |  |
| Quintile 5 (least deprived) | 1,175 | 585; 49.8% (46.9%-52.7%) | 590; 50.2% (47.3%-53.1%) |  |
| Missing | 46 | 14; 30.4% (17.7%-45.8%) | 32; 69.6% (54.2%-82.3%) |  |
| **Region (in England)** | | | | p<0.001 |
| London, South-East, East of England, and Thames Valley | 3,733 | 1,973; 52.9% (51.2%-54.5%) | 1,760; 47.1% (45.5%-48.8%) |  |
| West Midlands | 1,077 | 514; 47.7% (44.7%-50.8%) | 563; 52.3% (49.2%-55.3%) |  |
| North-West | 1,024 | 442; 43.2% (40.1%-46.3%) | 582; 56.8% (53.7%-59.9%) |  |
| East Midlands and South Yorkshire | 949 | 445; 46.9% (43.7%-50.1%) | 504; 53.1% (49.9%-56.3%) |  |
| South-West | 770 | 457; 59.4% (55.8%-62.8%) | 313; 40.6% (37.2%-44.2%) |  |
| Yorkshire and Humber | 699 | 305; 43.6% (39.9%-47.4%) | 394; 56.4% (52.6%-60.1%) |  |
| Northern | 480 | 206; 42.9% (38.4%-47.5%) | 274; 57.1% (52.5%-61.6%) |  |
| Wessex | 472 | 196; 41.5% (37.0%-46.1%) | 276; 58.5% (53.9%-63.0%) |  |
| **Clinical characteristics** | | | | |
| **CHD diagnosis types (in order of decreasing complexity)** | | | | p<0.001 |
| Hypoplastic left heart syndrome (HLHS) | 254 | 76; 29.9% (24.4%-36.0%) | 178; 70.1% (64.0%-75.6%) |  |
| Functionally univentricular heart (FUH) non-HLHS | 179 | 27; 15.1% (10.2%-21.2%) | 152; 84.9% (78.8%-89.8%) |  |
| Congenitally corrected transposition | 54 | 21; 38.9% (25.9%-53.1%) | 33; 61.1% (46.9%-74.1%) |  |
| Atrial isomerism with complex CHD | 118 | 74; 62.7% (53.3%-71.4%) | 44; 37.3% (28.6%-46.7%) |  |
| Common arterial trunk | 82 | 12; 14.6% (7.8%-24.2%) | 70; 85.4% (75.8%-92.2%) |  |
| Transposition of great arteries (TGA, all types) | 471 | 15; 3.2% (1.8%-5.2%) | 456; 96.8% (94.8%-98.2%) |  |
| Aortic stenosis (valvar, subvalvar) | 296 | 84; 28.4% (23.3%-33.9%) | 212; 71.6% (66.1%-76.7%) |  |
| Pulmonary atresia (intact septum or with VSD) | 167 | 27; 16.2% (10.9%-22.6%) | 140; 83.8% (77.4%-89.1%) |  |
| Tetralogy of Fallot (all types) | 647 | 79; 12.2% (9.8%-15.0%) | 568; 87.8% (85.0%-90.2%) |  |
| Double outlet ventricle (misc types) | 156 | 33; 21.2% (15.0%-28.4%) | 123; 78.8% (71.6%-85.0%) |  |
| Atrioventricular septal defect (AVSD, all types) | 558 | 173; 31.0% (27.2%-35.0%) | 385; 69.0% (65.0%-72.8%) |  |
| Totally anomalous pulmonary venous connection (TAPVC) | 148 | 18; 12.2% (7.4%-18.5%) | 130; 87.8% (81.5%-92.6%) |  |
| Aortic obstruction | 743 | 148; 19.9% (17.1%-23.0%) | 595; 80.1% (77.0%-82.9%) |  |
| Aortic arch malformations + vascular ring | 747 | 531; 71.1% (67.7%-74.3%) | 216; 28.9% (25.7%-32.3%) |  |
| Tricuspid valve abnormality (including Ebstein's) | 256 | 187; 73.0% (67.2%-78.4%) | 69; 27.0% (21.6%-32.8%) |  |
| Pulmonary stenosis | 487 | 196; 40.2% (35.9%-44.8%) | 291; 59.8% (55.2%-64.1%) |  |
| Mitral - aortic valve diseases nonobstructive | 253 | 177; 70.0% (63.9%-75.5%) | 76; 30.0% (24.5%-36.1%) |  |
| Pulmonary other group - valve non obstructive or pulmonary artery diseases | 194 | 156; 80.4% (74.1%-85.8%) | 38; 19.6% (14.2%-25.9%) |  |
| Isolated ventricular septal defect (VSD) | 2,750 | 2,031; 73.9% (72.2%-75.5%) | 719; 26.1% (24.5%-27.8%) |  |
| Other ungrouped CHDs | 644 | 473; 73.4% (69.9%-76.8%) | 171; 26.6% (23.2%-30.1%) |  |
| **Congenital non-cardiac comorbidity** |  |  |  | P=0.48^‡^ |
| Without comorbidity | 6,354 | 3,149; 49.6% (48.3%-50.8%) | 3,205; 50.4% (49.2%-51.7%) |  |
| With comorbidity (any type) | 2,850 | 1,389; 48.7% (46.9%-50.6%) | 1,461; 51.3% (49.4%-53.1%) |  |
| Genetic | 1,302 | 574; 44.1% (41.4%-46.8%) | 728; 55.9% (53.2%-58.6%) |  |
| Digestive system | 558 | 340; 60.9% (56.7%-65.0%) | 218; 39.1% (35.0%-43.3%) |  |
| Urinary | 337 | 161; 47.8% (42.3%-53.3%) | 176; 52.2% (46.7%-57.7%) |  |
| Limb | 223 | 153; 68.6% (62.1%-74.6%) | 70; 31.4% (25.4%-37.9%) |  |
| Nervous system | 225 | 137; 60.9% (54.2%-67.3%) | 88; 39.1% (32.7%-45.8%) |  |
| Oro-facial clefts/Eye/Ear, face and neck | 226 | 149; 65.9% (59.4%-72.1%) | 77; 34.1% (27.9%-40.6%) |  |
| Respiratory | 417 | 100; 24.0% (20.0%-28.4%) | 317; 76.0% (71.6%-80.0%) |  |
| Abdominal wall defects | 67 | 49; 73.1% (60.9%-83.2%) | 18; 26.9% (16.8%-39.1%) |  |
| Genital | 131 | 80; 61.1% (52.2%-69.5%) | 51; 38.9% (30.5%-47.8%) |  |
| Other anomalies / syndromes | 852 | 273; 32.0% (28.9%-35.3%) | 579; 68.0% (64.7%-71.1%) |  |
| **Antenatal detection** | | | | p=0.78 |
| With antenatal detection | 5,138 | 2,526; 49.2% (47.8%-50.5%) | 2,612; 50.8% (49.5%-52.2%) |  |
| Without antenatal detection | 4,066 | 2,012; 49.5% (47.9%-51.0%) | 2,054; 50.5% (49.0%-52.1%) |  |
| **Pre-term birth (<37 weeks' gestation)** | | | | p<0.001 |
| Pre-term birth | 2,339 | 1,454; 62.2% (60.2%-64.1%) | 885; 37.8% (35.9%-39.8%) |  |
| Full-term birth | 6,864 | 3,083; 44.9% (43.7%-46.1%) | 3,781; 55.1% (53.9%-56.3%) |  |
| *Sample size 1-5 (number suppressed).  ^†^ P-value for the chi-square test comparing pathway journeys in live birth babies among levels of demographic and clinical characteristics. Missing data were excluded from the analysis.  ^‡^ Test for comparing pathway journeys among live-born babies with any type of comorbidity vs. those without comorbidity. | | | | |

## Supplementary Table S11: Infant mortality rates for live-born babies with CHD by demographic and clinical characteristics.

|  | **Live birth with no cardiac intervention in infancy** | | **Live birth with cardiac intervention(s) in infancy** | |
| --- | --- | --- | --- | --- |
|  | **Number of patients** | **Infant death %** | **Number of patients** | **Infant death %** |
| **All patients** | 4,520 | 602 (13.3%) | 4,620 | 243 (5.3%) |
| **Demographic characteristics** | | | | |
| **Gender** | | | | |
| Boy | 2,281 | 302 (13.2%) | 2,678 | 131 (4.9%) |
| Girl | 2,237 | 299 (13.4%) | 1,941 | 112 (5.8%) |
| Missing | * | * | * | * |
| **IMD score (level of relative deprivation)** | | | | |
| Quintile 1 (most deprived) | 1,334 | 240 (18.0%) | 1,352 | 100 (7.4%) |
| Quintile 2 | 1,044 | 144 (13.8%) | 1,083 | 56 (5.2%) |
| Quintile 3 | 839 | 93 (11.1%) | 865 | 42 (4.9%) |
| Quintile 4 | 708 | 60 (8.5%) | 706 | 27 (3.8%) |
| Quintile 5 (least deprived) | 584 | 64 (11.0%) | 583 | 17 (2.9%) |
| Missing | 11 | * | 31 | * |
| **Region (in England)** | | | | |
| London, South-East, East of England, and Thames Valley | 1,966 | 227 (11.5%) | 1,737 | 71 (4.1%) |
| West Midlands | 513 | 88 (17.2%) | 558 | 41 (7.3%) |
| North-West | 438 | 80 (18.3%) | 579 | 41 (7.1%) |
| East Midlands and South Yorkshire | 445 | 60 (13.5%) | 500 | 35 (7.0%) |
| South-West | 455 | 45 (9.9%) | 311 | 13 (4.2%) |
| Yorkshire and Humber | 305 | 50 (16.4%) | 392 | 20 (5.1%) |
| Northern | 204 | 26 (12.7%) | 273 | 14 (5.1%) |
| Wessex | 194 | 26 (13.4%) | 270 | 8 (3.0%) |
| **Clinical characteristics** | | | | |
| **CHD diagnosis types (in order of decreasing complexity)** | | | | |
| Hypoplastic left heart syndrome (HLHS) | 76 | 71 (93.4%) | 178 | 48 (27.0%) |
| Functionally univentricular heart (FUH) non-HLHS | 27 | 16 (59.3%) | 152 | 11 (7.2%) |
| Congenitally corrected transposition | 21 | * | 33 | * |
| Atrial isomerism with complex CHD | 74 | 17 (23.0%) | 44 | 6 (13.6%) |
| Common arterial trunk | 12 | 11 (91.7%) | 70 | 6 (8.6%) |
| Transposition of great arteries (TGA, all types) | 15 | 9 (60.0%) | 452 | 26 (5.8%) |
| Aortic stenosis (valvar, subvalvar) | 84 | 13 (15.5%) | 209 | 11 (5.3%) |
| Pulmonary atresia (intact septum or with VSD) | 27 | 14 (51.9%) | 140 | 11 (7.9%) |
| Tetralogy of Fallot (all types) | 78 | 30 (38.5%) | 564 | 17 (3.0%) |
| Double outlet ventricle (Misc types) | 33 | 20 (60.6%) | 123 | 11 (8.9%) |
| Atrioventricular septal defect (AVSD, all types) | 173 | 52 (30.1%) | 380 | 37 (9.7%) |
| Totally anomalous pulmonary venous connection (TAPVC) | 18 | 13 (72.2%) | 130 | * |
| Aortic obstruction | 148 | 34 (23.0%) | 588 | 16 (2.7%) |
| Aortic arch malformations + vascular ring | 526 | 33 (6.3%) | 215 | 7 (3.3%) |
| Tricuspid valve abnormality (including Ebstein's) | 185 | 20 (10.8%) | 69 | * |
| Pulmonary stenosis | 195 | * | 286 | * |
| Mitral - aortic valve diseases nonobstructive | 177 | 27 (15.3%) | 74 | * |
| Pulmonary other group - valve non obstructive or pulmonary artery diseases | 156 | 12 (7.7%) | 38 | 0 (0%) |
| Isolated ventricular septal defect (VSD) | 2,023 | 142 (7.0%) | 706 | 11 (1.6%) |
| Other ungrouped CHDs | 472 | 63 (13.3%) | 169 | 11 (6.5%) |
| **Congenital non-cardiac comorbidity** |  |  |  |  |
| Without comorbidity | 3,132 | 240 (7.7%) | 3,171 | 125 (3.9%) |
| With comorbidity (any type) | 1,388 | 362 (26.1%) | 1,449 | 118 (8.1%) |
| Genetic | 574 | 173 (30.1%) | 721 | 51 (7.1%) |
| Digestive system | 340 | 113 (33.2%) | 218 | 20 (9.2%) |
| Urinary | 161 | 52 (32.3%) | 176 | 16 (9.1%) |
| Limb | 153 | 52 (34.0%) | 70 | 9 (12.9%) |
| Nervous system | 136 | 71 (52.2%) | 88 | 13 (14.8%) |
| Oro-facial clefts/Eye/Ear, face and neck | 149 | 57 (38.3%) | 77 | 14 (18.2%) |
| Respiratory | 100 | 20 (20.0%) | 313 | 35 (11.2%) |
| Abdominal wall defects | 49 | 20 (40.8%) | 18 | * |
| Genital | 80 | 20 (25.0%) | 51 | 9 (17.6%) |
| Other anomalies / syndromes | 273 | 43 (15.8%) | 576 | 58 (10.1%) |
| **Antenatal detection** |  |  |  |  |
| With antenatal detection | 2,511 | 464 (18.5%) | 2,596 | 179 (6.9%) |
| Without antenatal detection | 2,009 | 138 (6.9%) | 2,024 | 64 (3.2%) |
| **Pre-term birth (<37 weeks' gestation)** |  |  |  |  |
| Pre-term birth | 1,450 | 301 (20.8%) | 874 | 73 (8.4%) |
| Full-term birth | 3,070 | 301 (9.8%) | 3,746 | 170 (4.5%) |
| n=64 patients (non-intervened: 18; and intervened: 46) who had missing data in ONS or censored before the age of one year were removed from the analysis.  *Sample size 1-5 (number suppressed). | | | | |

## Supplementary Figure S1: Journey diagrams for fetuses and babies with functionally univentricular heart (FUH) non-HLHS, TGA, aortic stenosis, and pulmonary atresia.

Panel (a): functionally univentricular heart non-HLHS, (b): transposition of great arteries (TGA), (c): aortic stenosis, and (d): pulmonary atresia.

In our dataset, there were cases with severe CHD survived to age one without intervention. One possible reason is that some patients might have been misclassified due to coding quality, as we acknowledged in the limitations. However, there may be real existing patients who survived to one year without intervention. These cases are highly understudied, and we reported them in our study.


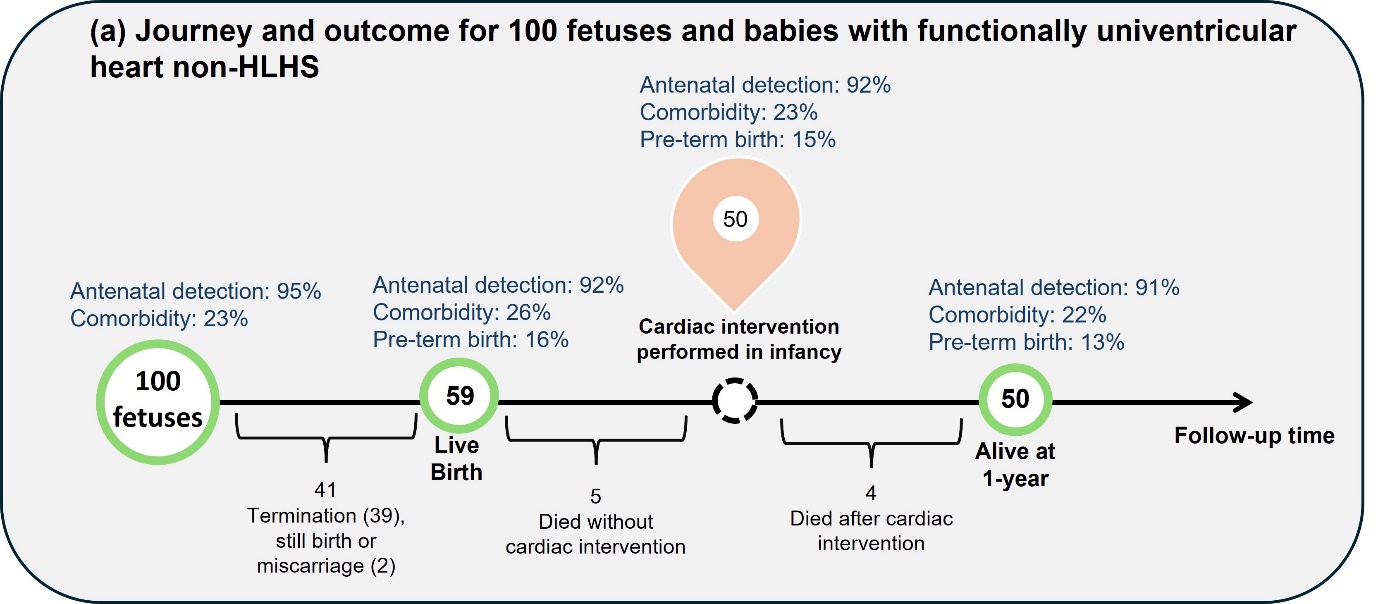


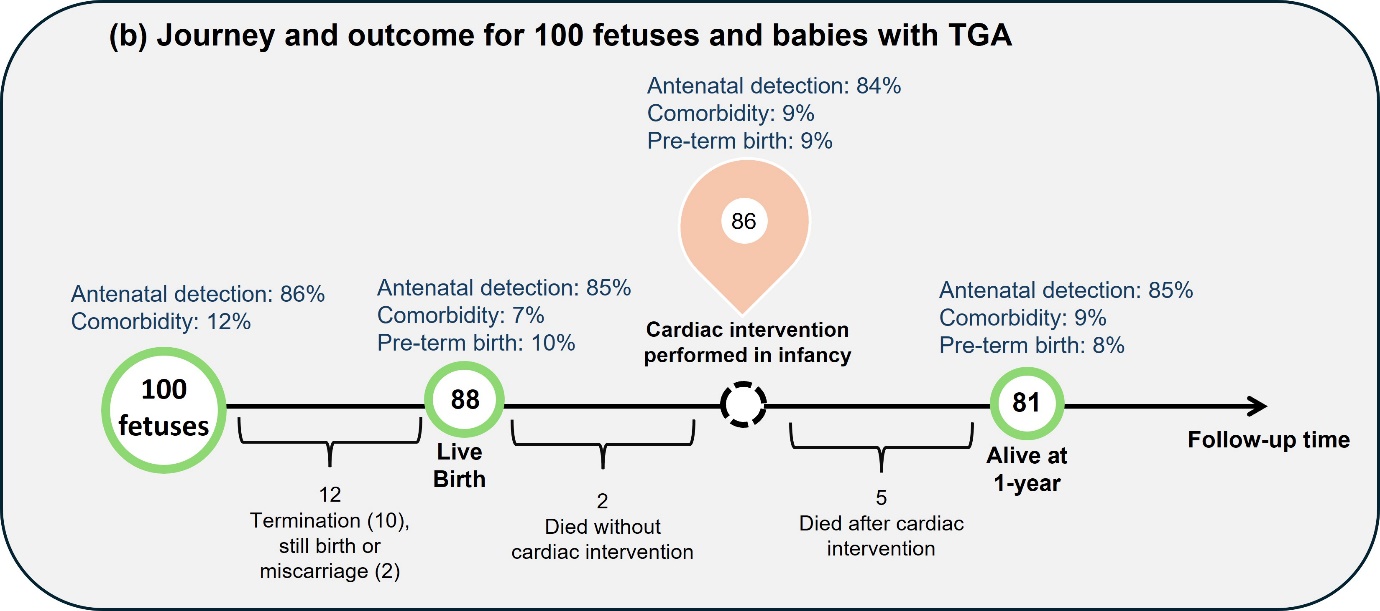


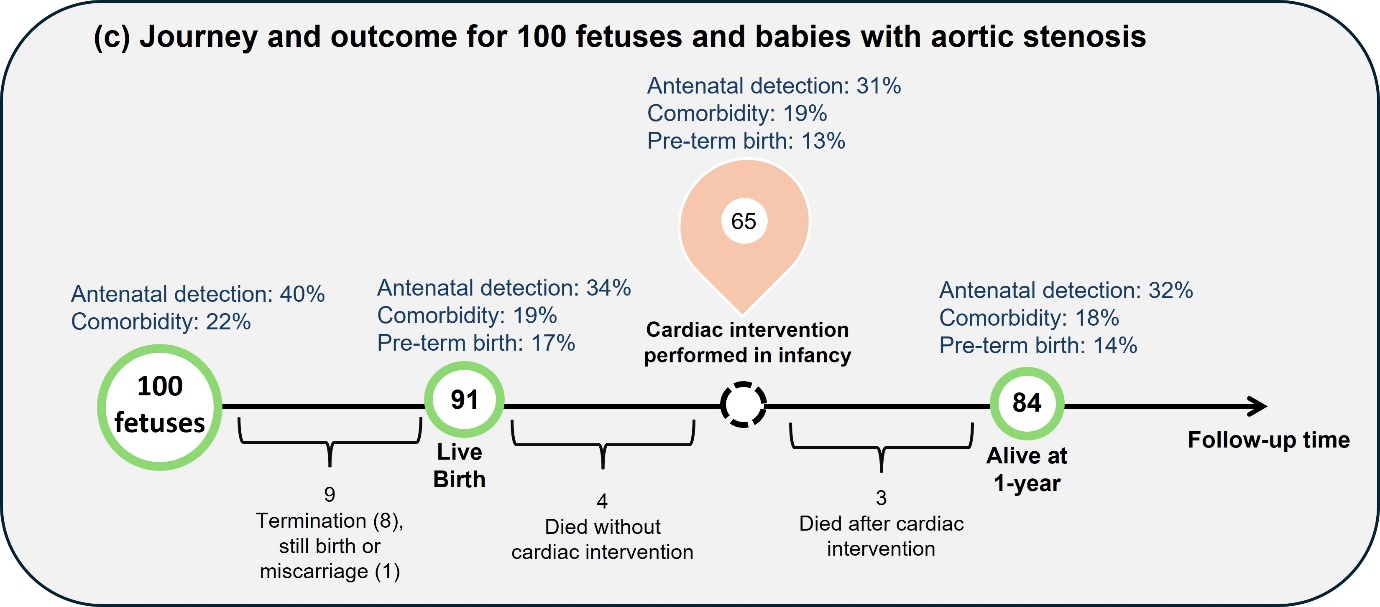


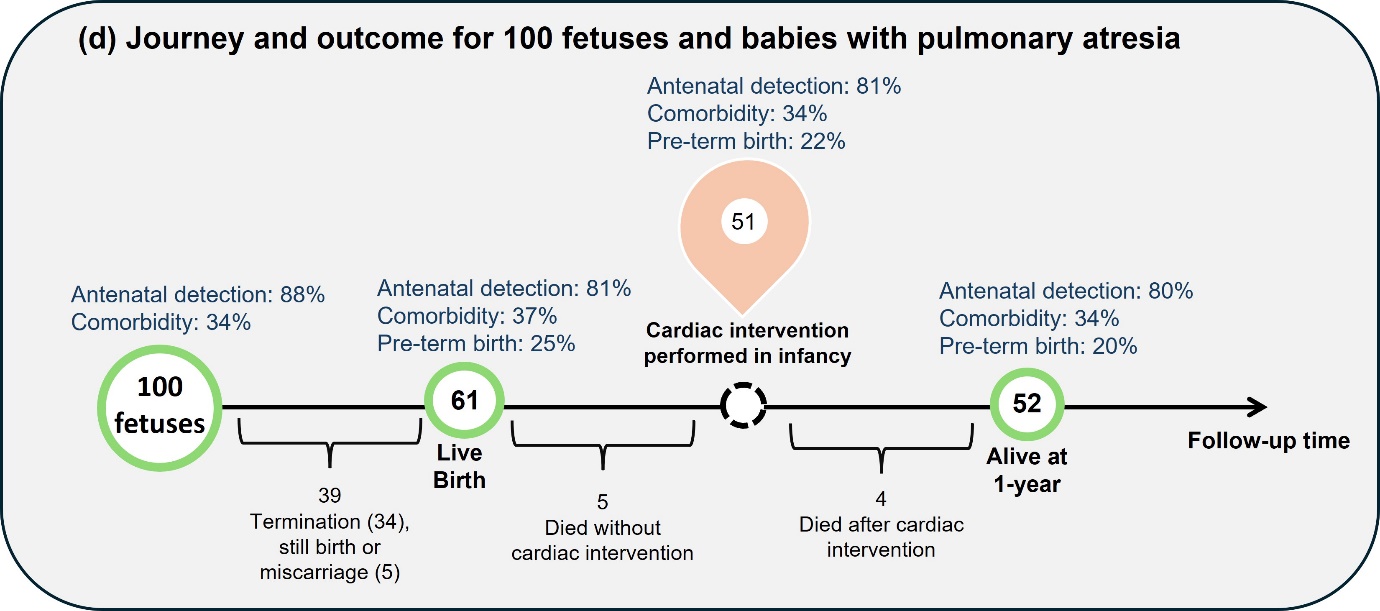

Supplement: online supplemental file 1 [file heartjnl-112-9-s001.docx]
